# Analysis of extracellular vesicle miRNA profiles in heart failure

**DOI:** 10.1111/jcmm.15251

**Published:** 2020-06-02

**Authors:** Jae Gyun Oh, Philyoung Lee, Ronald E. Gordon, Susmita Sahoo, Changwon Kho, Dongtak Jeong

**Affiliations:** ^1^ Cardiovascular Research Center Icahn School of Medicine at Mount Sinai New York NY USA; ^2^ Pathology Department Icahn School of Medicine at Mount Sinai New York NY USA; ^3^ Division of Applied Medicine School of Korean Medicine Pusan National University Republic of Korea

**Keywords:** bioinformatics, extracellular vesicle, heart failure, microRNA, microRNA array, microRNA control, miR‐676, U6

## Abstract

Extracellular vesicles (EVs) have recently emerged as an important carrier for various genetic materials including microRNAs (miRs). Growing evidences suggested that several miRs transported by EVs were particularly involved in modulating cardiac function. However, it has remained unclear what miRs are enriched in EVs and play an important role in the pathological condition. Therefore, we established the miR expression profiles in EVs from murine normal and failing hearts and consecutively identified substantially altered miRs. In addition, we have performed bioinformatics approach to predict potential cardiac outcomes through the identification of miR targets. Conclusively, we observed approximately 63% of predicted targets were validated with previous reports. Notably, the predicted targets by this approach were often involved in both beneficial and malicious signalling pathways, which may reflect heterogeneous cellular origins of EVs in tissues. Lastly, there has been an active debate on U6 whether it is a proper control. Through further analysis of EV miR profiles, miR‐676 was identified as a superior reference control due to its consistent and abundant expressions. In summary, our results contribute to identifying specific EV miRs for the potential therapeutic targets in heart failure and suggest that miR‐676 as a new reference control for the EV miR studies.

## 1 INTRODUCTION

A heart is a highly complex structure composed of multiple types of cellular and acellular tissues.^1^, ^2^ Of cardiac components, cardiomyocyte is a major contributor to cardiac composition and output.^1^ Non‐myocytes such as fibroblasts, leucocytes and endothelial cells serve as a spatial buffer and more importantly modulate cardiomyocyte functions in response to physiological and pathological stimuli. Thus, cardiomyocytes and non‐myocytes closely communicate to maintain cardiac homeostasis, and the imbalance in this communication causes cardiac diseases.^3^ Metabolites such as nitric oxide and reactive oxygen species and signalling molecules including extracellular matrix proteins, cytokines and growth factors are known to be classic mediators of intercellular communication.^3^, ^4^

Recently, extracellular vesicles (EVs) emerged as a mechanism underlying this intercellular communication. Although EVs had incipiently been considered as a means of cellular waste disposal, accumulating data recently indicated that genetic cargos such as mRNAs, microRNAs (miRs) and proteins are transported by EVs. Therefore, EVs are being re‐evaluated as a critical modifier of cellular functions.^5^ During last decade, tremendous efforts have been placed to understand the mechanism and the function of EVs in diverse fields of studies including cardiovascular researches. For example, EVs derived from stem cells or cardiac progenitor cells were generally beneficial,^6^, ^7^, ^8^, ^9^ whereas EVs from fibroblasts or endothelial cells exhibited deleterious effects on cardiac functions.^10^, ^11^, ^12^ In these previous studies, the origin of EVs was confined to in vitro cultured cells or blood plasma, but the role of EVs in cardiac tissues has been poorly investigated. Therefore, it is important to characterize in tissue EVs under pathological conditions. Recently, Loyer X. et al characterized the functions of EVs isolated from mouse hearts subjected to myocardial infarction.^13^ In this study, we further analysed the expression profiles and functions of miRNAs in EVs isolated from pressure overload‐induced failing hearts compared to normal hearts.

Apart from the characterization study, we also sought to identify an alternative internal reference for miR studies. In general, U6 is the most commonly used control for the normalization of miR expression. However, it is yet controversial whether U6 is a suitable control because variable expression patterns of U6 were observed in a number of studies.^14^, ^15^, ^16^, ^17^, ^18^, ^19^ Analysing the abundancy and stability of miR expression, U6 was confirmed as an appropriate control being consistently expressed throughout our samples. In addition, we identified miR‐676 as an alternative candidate as a control and observed that it exhibited a significant correlation with U6. Taken together, although a further validation is necessary in other tissues or organs, miR‐676 may serve as an alternative control for the EV miR studies in the heart.

In summary, this study analysed miR profiles in the cardiac EVs isolated from murine normal and failing hearts. Our results demonstrated that the cardiac EVs contain diverse miRs with contradictory functions. The results may reflect various cellular origins of EVs and dynamic alternations of miR expressions in these EVs during heart failure. Nevertheless, our study may contribute to understand intercellular communication between cardiac cells under the pathological condition and suggests EV miRs as potential biomarkers and therapeutic targets for the intervention of heart failure.

## 2 MATERIALS AND METHODS

### 2.1 Animal care and TAC

All procedures were approved by and performed in accordance with the Institutional Animal Care and Use Committee of the Mount Sinai School of Medicine. The investigation conformed to the Guide for the Care and Use of Laboratory Animals published by the US National Institutes of Health (NIH Publication No. 85‐23, revised 1996). Studies were conducted in male C57BL/6 mice aged 8 ~ 10 weeks (weight, 25 ~ 30 g) obtained from Jackson Laboratories. Transverse aortic constriction (TAC) was conducted as previously described with minor modifications.^10^ Briefly, mice were anesthetized with a solution mixture of 95 mg/kg ketamine and 5 mg/kg xylazine administered via intraperitoneal injection. The transverse aortic arch was ligated between the innominate and left common carotid arteries with an overlaid 27‐gauge needle. The needle was then immediately removed, leaving a discrete region of constriction. Two month after TAC operation, the mice showed less than 40% of fractional shortening in average which is typically considered as failing heart and included in this study (Figure S3). Sham‐operated mice underwent the same surgical procedures, except that the ligature was not tied.

### 2.2 EV isolation and purification

To isolate in tissue cardiac EVs from the heart tissue, Langendorff‐based isolation was applied as utilizing previous protocol to isolate in tissue EV from liver.^20^ Hearts were extracted from sham or TAC‐operated animals, and the aorta was retrogradely perfused at 37°C for 3 minutes with Tyrode buffer (137 mmol/L NaCl, 5.4 mmol/L KCl, 1 mmol/L MgCl_2_, 10 mmol/L glucose, 10 mmol/L HEPES [pH 7.4], 10 mmol/L 2, 3‐butanedione monoxime, and 5 mmol/L taurine) gassed with 100% O_2_. The enzymatic digestion was initiated by the addition of collagenase type B (300 U/mL; Worthington) and hyaluronidase (0.1 mg/mL; Worthington) to the perfusion solution. After 20 minutes of digestion, the heart tissue was removed, cut into several chunks and gently pipetted for 2 minutes in Tyrode buffer with 5% BSA. The mixtures of cell, extracellular matrix and EVs were then separated by several centrifugations according to a previously described protocol.^21^ EVs were pelleted by ultracentrifugation at 100 000 × *g* at 4°C for 90 minutes and resuspended in 200 μL of PBS solution.

### 2.3 Transmission Electron microscopy for EVs with negative staining

EVs from fresh ventricles were collected as described above and pre‐fixed with 2% paraformaldehyde (PFA) for 30 minutes. Deposit 5 μL resuspended pellets on Formvar‐carbon‐coated EM grid. Add 10 μL of 2% uranyl acetate on the grid and incubate for another 10 minutes. After thorough washes, store grid in the grid box at room temperature and dark until imaging. Samples were viewed under a transmission electron microscope (HITACHI H‐7650, Japan) operated at 80 kV. Images were taken at 10K, 20K and 30K‐folds magnification.

### 2.4 Western blot of EV markers and Coomassie blue stain

50 μL of EV samples from Sham and TAC EV was directly mixed with 5x SDS sample buffer, boiled at 95°C for 3 minutes and then separated on a SDS‐PAGE gel. The total loaded protein amount was calculated by the intensity of Coomassie blue stain. The membrane was blocked in 5% skim milk solution and incubated overnight with an antibody directed against hnRNA A2B1 (Ab6102, Abcam, 1:5000), CD63 (Ab1318, Abcam, 1:1000), Flotillin 1 (Ab133497, Abcam, 1:10 000), TSG101 (Ab83, Abcam, 1:1000), H3 (Ab1791, Abcam, 1:1000) and SERCA2a (custom antibody from 21st Century Biochemicals, 1:3000). The membrane was then incubated with a horseradish peroxidase‐conjugated secondary antibody (Sigma) and developed with Western Lighting chemiluminescence reagent (PerkinElmer).

### 2.5 Nanoparticle tracking analysis

Samples were loaded into the assembled sample chamber of a NanoSight LM10. 60‐second video images were acquired by a Hamamatsu C11440 ORCA‐Flash 2.8 digital camera and analysed by NanoSight NTA 2.3 software.

### 2.6 Extracellular vesicle microRNA isolation and microRNA array profiling

100 μL of EV samples from Sham and TAC EV was used to extract RNA for microRNA array profiling and validation using qRT‐PCR. Total EV RNA was isolated with mirVana miRNA Isolation Kit (Ambion). The RNA samples were sent, and the microarray procedure was carried out at Exiqon Vedbaek, Denmark, as followed the previous description.^22^ Briefly, the samples were labelled using the miRCURY LNA™ microRNA Hi‐Power Labeling Kit, Hy3™/Hy5™ and hybridized on the miRCURY LNA™ microRNA Array (7th Gen).

### 2.7 qRT‐PCR

Total RNA was isolated with mirVana miRNA Isolation Kit (Ambion). Reverse transcription was performed using qScript microRNA cDNA Synthesis Kit (Quanta). PCR was performed using an ABI PRISM Sequence Detector System 7500 (Applied Biosystems) with SYBR Green (Quanta) as the fluorescent dye and ROX (Quanta) as the passive reference dye. The relative amount of each gene to U6 snRNA was used to quantify cellular RNA. The primers used for qRT‐PCR were as follows:

miR‐92b (forward): 5′‐TAT TGC ACT CGT CCC GGC CTC C‐3′

miR‐139 (forward): 5′‐TCT ACA GTG CAC GTG TCT CCA G‐3′

miR‐328 (forward): 5′‐CTG GCC CTC TCT GCC CTT CCG T‐3′

miR‐331 (forward): 5′‐CTA GGT ATG GTC CCA GGG ATC C‐3′

miR‐345 (forward): 5′‐GCT GAC CCC TAG TCC AGT GCT T‐3′

miR‐378a (forward): 5′‐ACT GGA CTT GGA GTC AGA AGG‐3′

miR‐490 (forward): 5′‐CCA TGG ATC TCC AGG TGG GT‐3′

miR‐655 (forward): 5′‐ACC AGG AGG CTG AGG TCC CT‐3′

miR‐767 (forward): 5′‐TGC ACC ATG GTT GTC TGA GCA‐3′

miR‐874 (forward): 5′‐CTG CCC TGG CCC GAG GGA CCG A‐3′

miR‐99b (forward): 5′‐CAC CCG TAG AAC CGA CCT TGC G‐3′

miR‐124 (forward): 5′‐TAA GGC ACG CGG TGA ATG CC‐3′

miR‐184 (forward): 5′‐TGG ACG GAG AAC TGA TAA GGG T‐3′

miR‐200b (forward): 5′‐TAA TAC TGC CTG GTA ATG ATG A‐3′

miR‐302a (forward): 5′‐TAA GTG CTT CCA TGT TTT GGT GA‐3′

miR‐411 (forward): 5′‐TAG TAG ACC GTA TAG CGT ACG‐3′

miR‐455 (forward): 5′‐GCA GTC CAC GGG CAT ATA CAC‐3′

miR‐676 (forward): 5′‐CCG TCC TGA GGT TGT TGA GCT‐3′

miR‐300 (forward): 5′‐TAT GCA AGG GCA AGC TCT CTT C‐3′

miR‐204 (forward): 5′‐TTC CCT TTG TCA TCC TAT GCC T‐3′

miR‐453 (forward): 5′‐AGG TTG CCT CAT AGT GAG CTT GCA‐3′

miR‐3085 (forward): 5′‐TCT GGC TGC TAT GGC CCC CTC‐3′

miR‐146a (forward): 5′‐TGA GAA CTG AAT TCC ATG GGT T‐3′

Reverse primer: PerfeCTa^®^ Universal PCR Primer (Quanta)

U6 (forward): 5′‐CTC GCT TCG GCA GCA CA‐3′

U6 (reverse): 5′‐AAC GCT TCA CGA ATT TGC GT‐3′

### 2.8 Bioinformatics analysis

The global UP and DOWN cardiac microRNA target genes were generated using miTarBase (Http://mitarbase.mbc.nctu.edu.tw). Bioinformatics analyses (Gene Ontology) were performed using the UniProt database and Human Protein Reference Database.^23^ Protein‐protein interactions analyses by STRING 11.0 database with medium confidence STRING annotated interactions (STRING score > 0.4) for the bait proteins.^24^ Cytoscape software (Ver 3.6.1; http://www.cytoscape.org/) was used to visualize the network.

### 2.9 Statistical analysis

Where appropriate, the data are expressed as means ± s.e.m. Comparisons of the group means were made by using Student's *t* test or one‐way ANOVA with a Bonferroni post‐test analysis. Correlation test was performed with the Pearson's correlation coefficient measure. A *P*‐value of <.05 was considered to be statistically significant.

## 3 RESULTS

### 3.1 Cardiac EVs from normal and failing hearts have distinctive physical and molecular signatures

**Figure 1:**
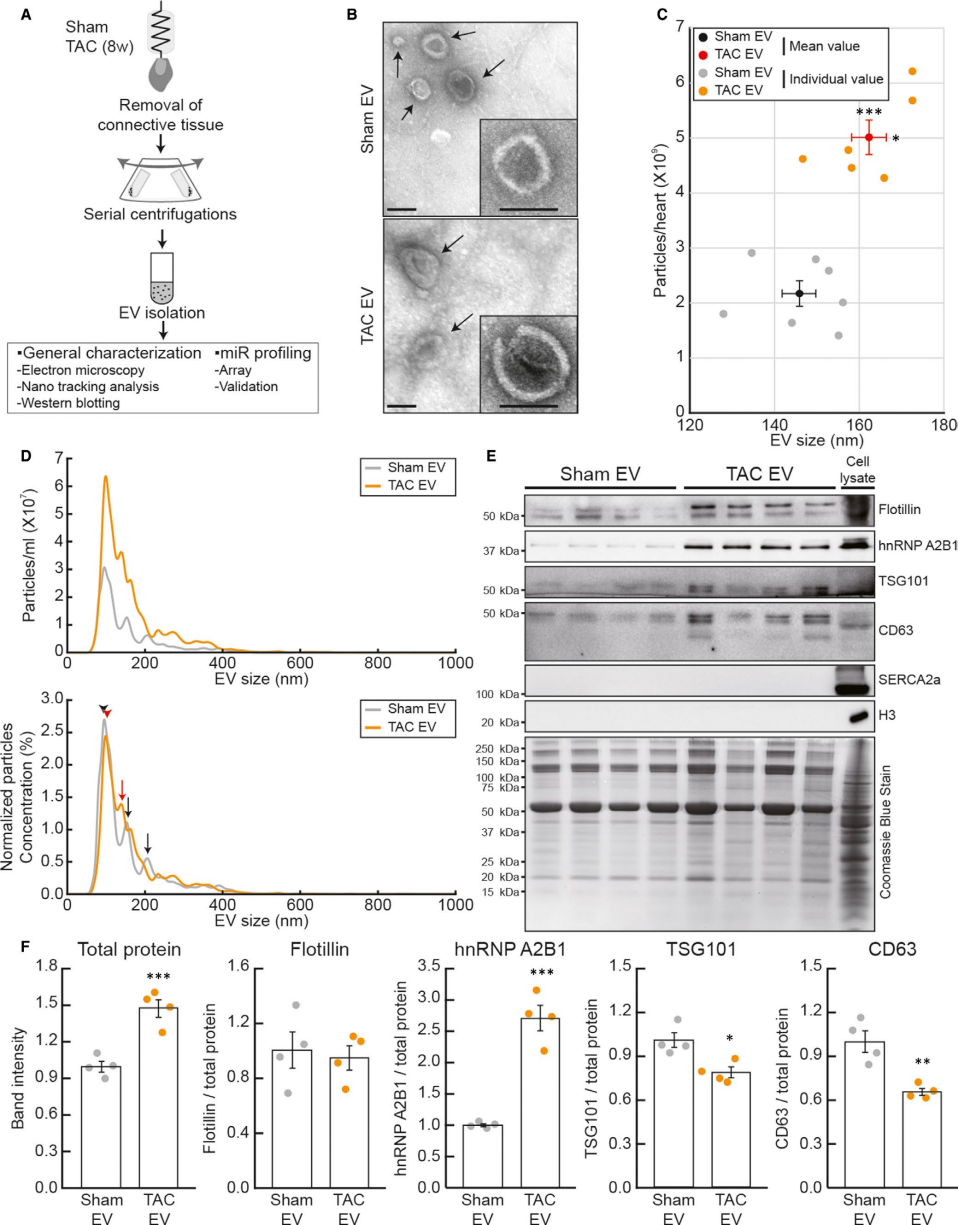
Characterization of in tissue cardiac EVs. A, Schematic procedure for the EV isolation. B, Representative images of electron microscopy. The bar indicates 100 nm. Arrows indicate EVs. C, Average densities and sizes of Sham and TAC EV as analysed by NTA. n = 6‐7. D, Ensemble averages from several identical size distributions of Sham (n = 7) and TAC EV (n = 6). Upper: Absolute particle numbers per 1 mL, Lower: Relative concentration normalized to total nanoparticle concentrations. Arrow heads and arrows indicate the primary peak and the second or third peak, respectively. E, Representative images of Western blot and (F) quantified data. Sham EV, TAC EV and NTA indicate EVs from normal hearts, EVs from failing hearts and nanoparticle tracking analysis, respectively. *P < .05, **P < .01, ***P < .001 versus Sham EV, as determined by Student's t test. Data are presented as mean ± s.e.m

Pressure overload‐induced heart failure was generated in murine hearts by applying transverse aortic constriction (TAC). The cardiac EVs were isolated from normal (Sham EVs) and failing (TAC EVs) hearts using the Langendorff‐based isolation followed by purification through multiple rounds of ultracentrifugations (Figure 1A). The isolated EVs were negatively stained and visualized by electron microscopy with 30 000‐fold magnification. Morphological analysis revealed that EVs showed a typical cup‐shaped structure with a diameter of 70 ~ 160 nm and that TAC EVs tended to be larger than Sham EVs (Figure 1B). Nanoparticle tracking analysis (NTA) was performed to precisely measure the concentration and sizes of EVs. Failing hearts secrete ~5 × 10^9^ EVs per heart, whereas normal hearts release ~2 × 10^9^ EVs per heart (Figure 1C). Average size of TAC EVs was significantly larger than that of Sham EVs, which is consistent with the electron microscopic data (Figure 1D upper panel). Size distribution profiles of EVs indicated that the majority of EVs were 100 nm in size in both Sham and TAC groups. Two additional groups of Sham EVs were 150 nm and 200 nm in size. On the other hand, one group of 130 nm in size was additionally observed in TAC EVs (Figure 1D, black arrow; Sham EVs, red arrow; TAC EVs). Furthermore, Sham and TAC EVs exhibited a significant difference in their protein contents. Same volumes of EV samples (50 μL, a quarter of the total EVs from a heart) were loaded in a SDS‐PAGE gel and quantified by Coomassie blue staining. In line with NTA results, 50% more proteins were detected in TAC EVs (Figure 1E and F). As shown in Figure 1F, several EV markers were analysed by Western bolt analysis. The Flotillin level was not altered between Sham and TAC EVs, but hnRNP A2B1 expression was significantly increased in TAC EVs. On the other hand, TSG101 and CD63 expressions were substantially reduced in TAC EVs (Figure 1F). Taken together, the cardiac EVs from normal and failing hearts showed distinctive features in their physical and molecular properties.

### 3.2 microRNA profiles of cardiac EVs and validation

**Figure 2:**
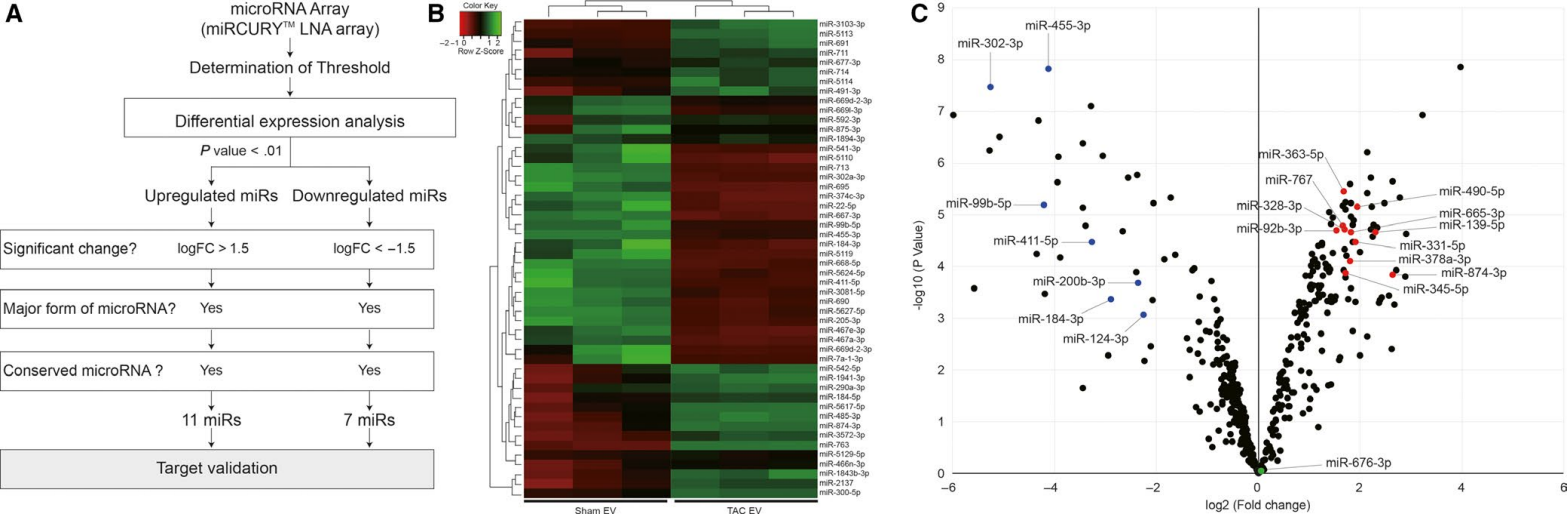
microRNA array results of tissue cardiac EVs. A, A scheme of entire procedure to identify differentially expressed microRNAs. B, Heat map and hierarchical clustering. The clustering was performed on the top 50 miRs with highest standard deviation. C, Volcano plot depicting the fold changes (*X*‐axis) and *P*‐value (*Y*‐axis) in miR expression levels between Sham and TAC EV. Coloured points refer significantly up‐regulated (Red), down‐regulated (Blue) or unchanged (Green) microRNAs

**Figure 3:**
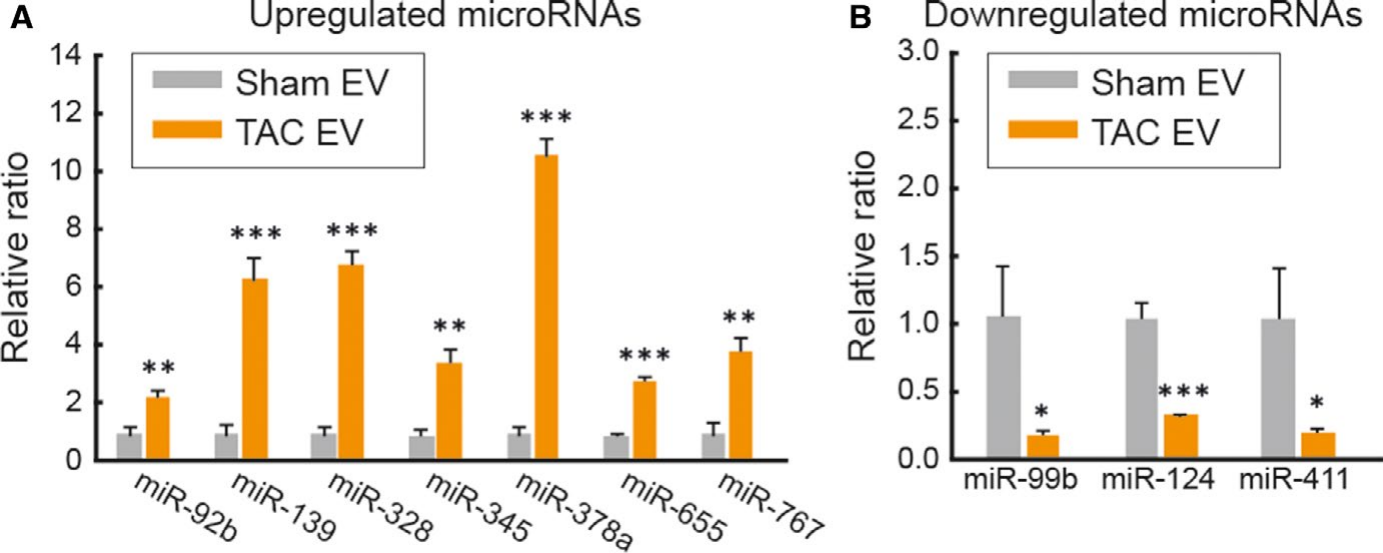
Validation of microRNA expression profiles by qRT‐PCR. The levels of the up‐regulated (A) or down‐regulated (B) miRs were quantified by qRT‐PCR. The relative levels of each microRNA were normalized to the U6 snRNA. n = 4. *P < .05, **P < .01, ***P < .001 versus Sham EV, as determined by one‐way ANOVA. Data are presented as mean ± s.e.m

To further characterize cardiac EVs, microRNA (miR) expression profiles were explored. Cardiac EVs were isolated from three normal and three failing hearts. All EV preparations were immediately subjected to quality control and miRNA array analyses that were conducted using the 7th generation of miRCURY™ LNA array system (Exiqon). Of the 1,195 miRs displayed on the array, 731 miRs were eliminated due to a lack of significant signal intensity. The remaining 464 miRs were then sorted out by criteria indicated in Figure 2A (Table S1). The heat map diagram demonstrated a clear separation of miR expressions in Sham and TAC EVs (Figure 2B). Compared to Sham EVs, 107 miRs were up‐regulated (23%) and 47 miRs were down‐regulated (10%) in TAC EVs (Figure 2C). miRs with p‐values higher than 0.05 were considered as ‘not altered’ (67%). The 154 miRs differentially expressed on the array were further validated for their abundancy using qRT‐PCR. Consequently, we identified seven up‐regulated (miR‐378a, miR‐665, miR‐139, miR‐345, miR‐328, miR‐767 and miR‐92b) and three down‐regulated (miR‐99b, miR‐124 and miR‐411) miRs (Figure 3A and B).

### 3.3 Potential target prediction for the identified miRs

**Figure 4:**
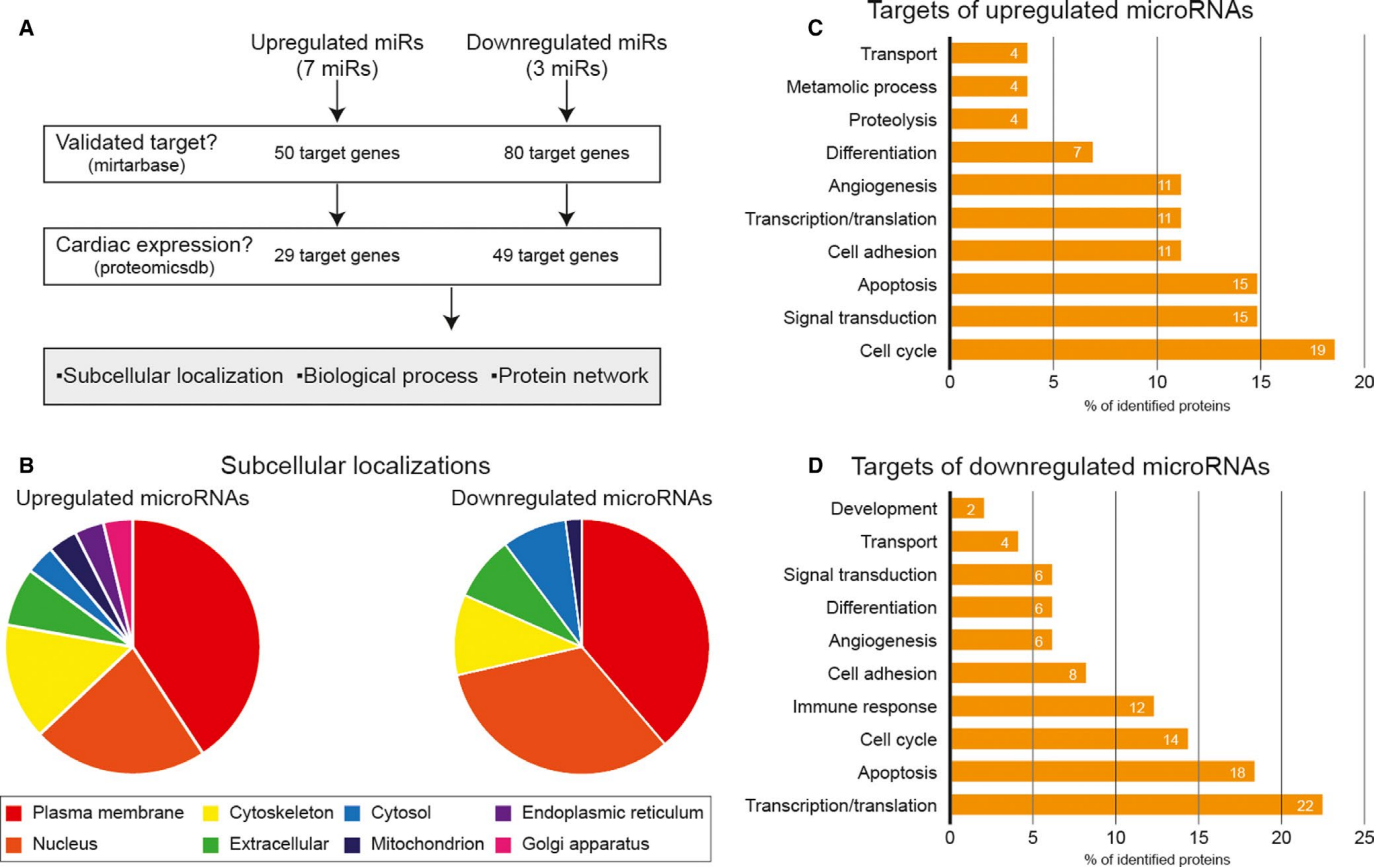
Proteomics analysis of the putative microRNA target genes. A, Analyses were conducted based on miRNA database (miRTarBase; http://mirtarbase.mbc.nctu.edu.tw) and protein database (ProteomicsDB; www.proteomicsdb.org). Target proteins were displayed by their subcellular localization (B) and Gene Ontology (GO) categories (C and D) using the STRING database (https://string‐db.org/)

**Figure 5:**
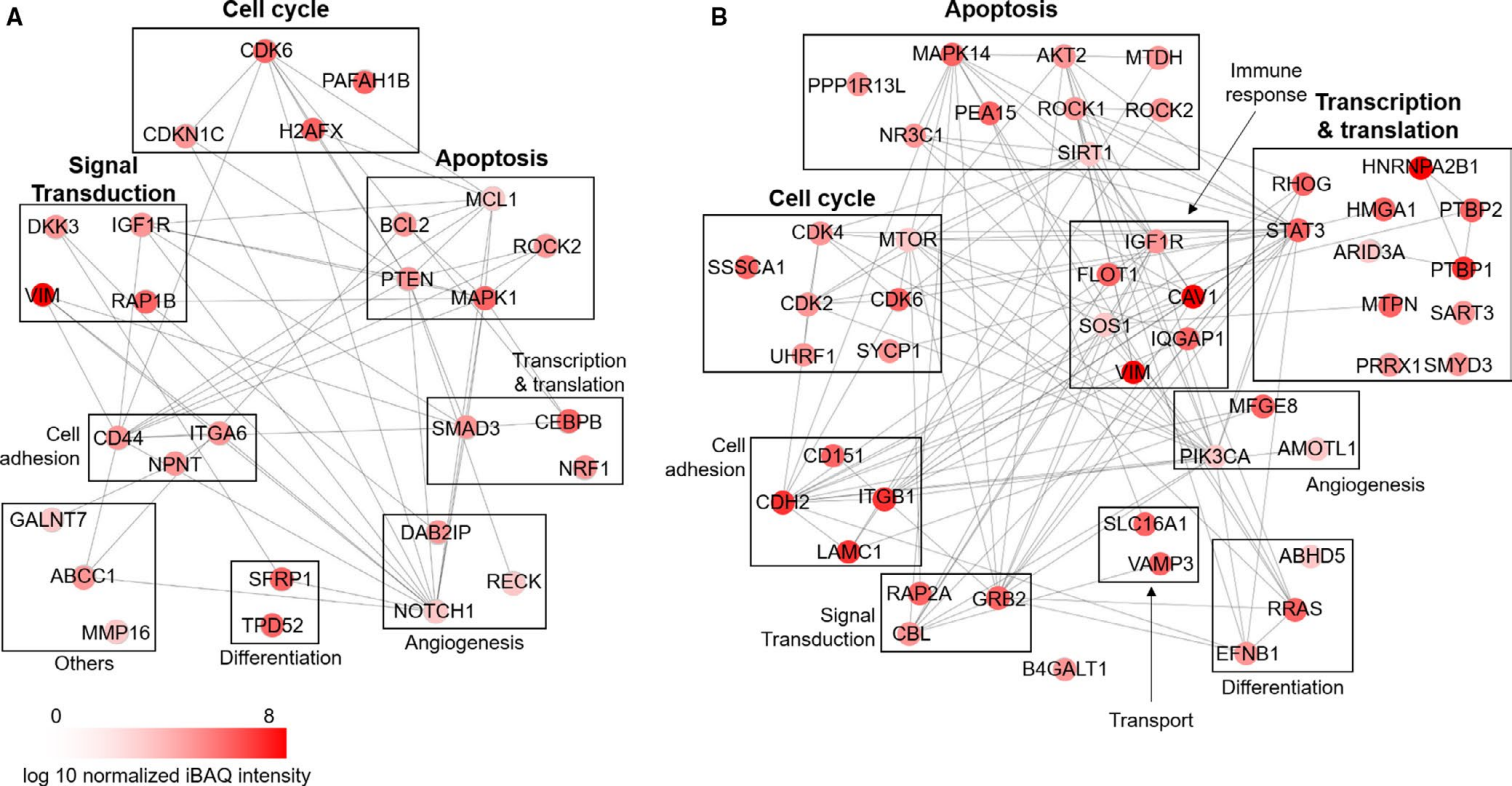
In silico protein‐protein interaction map for putative microRNA targets. The protein‐protein maps were constructed using STRING 11.0 database and Cytoscape. The putative molecular targets by up‐regulated microRNAs (A) and down‐regulated microRNAs (B). The iBAQ intensity represents the expression level of proteins in the heart

To investigate the roles of the identified miRs under pathological conditions, bioinformatics analyses were performed as shown in Figure 4A (Tables 1 and 2). First, putative targets listed in miRTarBase (http://mirtarbase.mbc.nctu.edu.tw) as validated by both reporter assay and Western blot analysis were selected. Second, the selected targets were further sorted out according to their tissue distribution as reported in the proteomics database (www.proteomicsdb.org). Through these two subsequent analyses, 29 putative targets associated with the seven up‐regulated miRs and 49 putative targets associated with the three down‐regulated miRs were identified. Lastly, these putative targets were categorized based on their subcellular localization, biological process and protein‐protein interactions (Tables 3 and 4). The analysis of subcellular localization revealed that miRs of TAC EVs appeared to target proteins predominantly located in the plasma membrane, nucleus and cytoskeleton (Figure 4B). Functional analysis suggested that target proteins for the up‐regulated miRs were involved in cell cycle (19%), signal transduction (15%) and apoptosis (15%). On the other hand, intracellular targets for the down‐regulated miRs were predicted to contribute to transcription/translation (22%), apoptosis (18%) and cell cycle (14%) (Figure 4 and D). The protein‐protein interaction map generated by the STRING database (https://string‐db.org/) is shown in Figure 5. The most frequently matched targets controlled by the up‐regulated miRs in TAC EVs were NOTCH, CD44 and PTEN. Meanwhile, targets modulated by the down‐regulated miRs were CDH2, GRB2 and ITGB1. Taken together, these results suggest that miRs in TAC EVs are indeed closely related to the essential biological processes.

**Table 1:** Up‐regulated miRs in TAC EV and their targets

Name	*P*.Value		Targets
	Microarray	qRT‐PCR	
miR‐767‐5p	1.7E‐05	0.002	
miR‐328‐3p	1.9E‐05	1.4E‐06	ABCG2, CD44, H2AFX, KCNH2, MMP16, PLCE1, SFRP1
miR‐92b‐3p	2.0E‐05	0.004	CDKN1C, CEBPB, DAB2IP, DKK3, ITGA6, NLK, NOX4, PTEN, RECK, SLC15A1, SMAD3
miR‐139‐5p	2.2E‐05	7.6E‐05	ADGRL4, BCL2, CXCR4, FOXO1, IGF1R, IRS1, JUN, MCL1, MET, MMP11, NOTCH1, NR5A2, OIP5, PAFAH1B1, RAP1B, ROCK2, TPD52, WNT1
miR‐665‐3p	2.2E‐05	2.6E‐05	
miR‐378a‐3p	8.0E‐05	1.0E‐07	CDK6, GALNT7, IGF1R, MAPK1, NPNT, NRF1, PGR, RUNX1, TGFB2, VEGFA, VIM, WNT10A
miR‐345‐5p	1.3E‐04	0.001	ABCC1, CDKN1A

**Table 2:** Down‐regulated miRs in TAC EV and their targets

Name	*P*.Value		Targets
	Microarray	qRT‐PCR	
miR‐99b‐5p	6.4E‐06	0.041	ARID3A, IGF1R, MFGE8, MTOR, RAVER2
miR‐124‐3p	8.7E‐04	1.1E‐04	ABHD5, ADIPOR2, AHR, AKT2, AMOTL1, AR, B4GALT1, CAMTA1, CAV1, CBL, CCL2, CCNA2, CCND2, CD151, CDH2, CDK2, CDK4, CDK6, CEBPA, CLOCK, DLX5, E2F6, EFNB1, EGR1, EZH2, FLOT1, FOXA2, HMGA1, HNRNPA2B1, HOTAIR, IL6R, IQGAP1, ITGB1, JAG1, LAMC1, MAPK14, MTDH, MTPN, MYO10, NFATC1, NFKBIZ, NR3C1, NR3C2, PEA15, PIK3CA, PNPLA2, PPP1R13L, PRRX1, PTBP1, PTBP2, RAB38, RAP2A, RHOG, ROCK1, ROCK2, RRAS, SART3, SIRT1, SLC16A1, SMOX, SMYD3, SOS1, SPHK1, SSSCA1, STAT3, SYCP1, TRIB3, UHRF1, VAMP3, VIM
miR‐411‐5p	3.3E‐05	0.048	GRB2

**Table 3:** Putative targets of the up‐regulated miRs and their localization, biological process and cardiac expression

Up‐regulated microRNAs					
Target Genes	Primary Localization	Primary Biological Process	Cardiac Expression	Expression in Failing Heart	Reference
miR‐92b‐3p					
CDKN1C	Nucleus	Cell cycle	4.36	Down	[53]
CEBPB	Nucleus	Transcription and translation	4.63	Down	[55]
DAB2IP	Plasma membrane	Angiogenesis	3.74	No study	
DKK3	Extracellular	Signal transduction	4.03	Down	[54]
ITGA6	Plasma membrane	Cell adhesion	4.35	No study	
PTEN	Plasma membrane	Apoptosis	4.43	Down	[52]
RECK	Plasma membrane	Angiogenesis	3.34	No study	
SMAD3	Nucleus	Transcription and translation	4.17	Up	[59]
miR‐139‐5p					
BCL2	Cytosol	Apoptosis	3.94	Down	[56]
IGF1R	Plasma membrane	Signal transduction	3.98	Up	[41]
MCL1	Mitochondrion	Apoptosis	3.42	Down	[57]
NOTCH1	Nucleus	Angiogenesis	2.97	Up	[60]
PAFAH1B1	Cytoskeleton	Cell cycle	5.44	No study	
RAP1B	Plasma membrane	Signal transduction	5.47	No study	
ROCK2	Plasma membrane	Apoptosis	4.38	Up	[42]
TPD52	Endoplasmic reticulum	Differentiation	4.97	No study	
miR‐328‐3p					
CD44	Plasma membrane	Cell adhesion	4.3	Up	[61]
H2AFX	Nucleus	Cell cycle	4.6	No study	
MMP16	Plasma membrane	Proteolysis	3.08	No study	
SFRP1	Plasma membrane	Differentiation	4.88	No change	
miR‐345‐5p					
ABCC1	Plasma membrane	Transport	3.66	No study	
miR‐378a‐3p					
CDK6	Cytoskeleton	Cell cycle	5.01	No study	
GALNT7	Golgi apparatus	Metabolic process	2.89	No study	
IGF1R	Plasma membrane	Signal transduction	3.98	Up	[41]
MAPK1	Cytoskeleton	Apoptosis	5.14	Up	[35]
NPNT	Extracellular	Cell adhesion	4.05	No study	
Nrf1	Nucleus	Transcription and translation	4.29	Up	[62]
VIM	Cytoskeleton	Signal transduction	7.38	Up	[47]

**Table 4:** Putative targets of the down‐regulated miRs and their localization, biological process and cardiac expression

Down‐regulated microRNAs					
Target Genes	Primary Localization	Primary Biological Process	Cardiac Expression	Expression in Failing Heart	Reference
miR‐99b‐5p					
ARID3A	Nucleus	Transcription and translation	3.08	No Study	
IGF1R	Plasma membrane	Immune response	3.98	Up	[41]
MFGE8	Cytoskeleton	Angiogenesis	4.51	Down	[63]
MTOR	Nucleus	Cell cycle	3.48	up	[46]
miR‐124‐3p					
ABHD5	Cytosol	Differentiation	2.66	Down	[64]
AKT2	Nucleus	Apoptosis	3.87	No Study	
AMOTL1	Cytosol	Angiogenesis	2.88	No Study	
B4GALT1	Plasma membrane	Development	3.79	No Study	
CAV1	Plasma membrane	Immune response	6.78	Down	[65]
CBL	Golgi apparatus	Signal transduction	3.59	Up	[50]
CD151	Plasma membrane	Cell adhesion	4.82	Controversial	
CDH2	Plasma membrane	Cell adhesion	5.96	Up	[43]
CDK2	Nucleus	Cell cycle	4.34	UP	[48]
CDK4	Nucleus	Cell cycle	4.11	No change	[66]
CDK6	Nucleus	Cell cycle	5.01	No Study	
EFNB1	Plasma membrane	Differentiation	4.42	No study	
FLOT1	Plasma membrane	Immune response	5.34	No Study	
HMGA1	Nucleus	Transcription and translation	5.49	Down	[67]
HNRNPA2B1	Extracellular	Transcription and translation	6.57	No Study	
IQGAP1	Plasma membrane	Immune response	4.56	No Study	
ITGB1	Plasma membrane	Cell adhesion	5.68	No Study	
LAMC1	Extracellular	Cell adhesion	6.25	No Study	
MAPK14	Nucleus	Apoptosis	4.97	Up	[44]
MTDH	Nucleus	Apoptosis	4.25	No Study	
MTPN	Cytoskeleton	Transcription and translation	5.6	No Study	
NR3C1	Nucleus	Apoptosis	4.26	No Study	
PEA15	Cytoskeleton	Apoptosis	5.43	No Study	
PIK3CA	Cytosol	Angiogenesis	2.89	Up	[51]
PPP1R13L	Extracellular	Apoptosis	4.06	No Study	
PRRX1	Nucleus	Transcription and translation	4.03	No Study	
PTBP1	Nucleus	Transcription and translation	5.69	No Study	
PTBP2	Nucleus	Transcription and translation	4.98	No Study	
RAP2A	Plasma membrane	Signal transduction	4.3	No Study	
RHOG	Plasma membrane	Transcription and translation	5.07	No Study	
ROCK1	Cytoskeleton	Apoptosis	4.25	UP	[42]
ROCK2	Plasma membrane	Apoptosis	4.38	UP	[42]
RRAS	Plasma membrane	Differentiation	5.33	No Study	
SART3	Nucleus	Transcription and translation	4.07	Up	[45]
SIRT1	Nucleus	Apoptosis	3.37	Up	[49]
SLC16A1	Plasma membrane	Transport	5.16	No Study	
SMYD3	Nucleus	Transcription and translation	3.99	No Study	
SOS1	Cytosol	Immune response	3.31	No Study	
SSSCA1	Extracellular	Cell cycle	4.74	No Study	
STAT3	Nucleus	Transcription and translation	4.91	Up	[40]
SYCP1	Nucleus	Cell cycle	4.27	No Study	
UHRF1	Nucleus	Cell cycle	4.04	No Study	
VAMP3	Plasma membrane	Transport	4.88	No Study	
VIM	Cytoskeleton	Immune response	7.38	Up	[47]
miR‐411‐5p					
GRB2	Plasma membrane	Signal transduction	5.19	No Study	

### 3.4 Identification of an alternative control for miR expression

**Figure 6:**
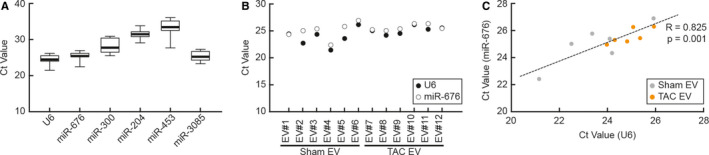
Identification of internal microRNA control. A, The Ct values of U6 and microRNA candidates as an internal control were quantified by qRT‐PCR. n = 12, data are presented as mean ± s.e.m. B, The Ct values of U6 and miR‐676 in entire 12 samples were presented. C, The Pearson correlation coefficient was used to measure the strength of a linear association between U6 and miR‐676 expressions. *R* = 0.825, *P* = .001

There is no clear consensus on suitable controls for relative miR quantification in circulating miRs, although U6 has been considered as an endogenous normalization control.^14^, ^15^, ^16^, ^17^, ^18^, ^19^ In this study, we tested whether U6 is an adequate control for the miR expression. U6 expression was shown to be abundant, stable and not significantly different across all 12 EV samples (Figure 6A). Therefore, U6 expression was used to normalize the qRT‐PCR data presented in Figure 3. To further support and strengthen our results, we searched out potential candidates for more reliable controls that showed minimal differences in expressions between groups yet with strong signal intensity. Initial screening led us to identify five miRs, miR‐676, miR‐300, miR‐204, miR‐453 and miR‐3085. Those were thoroughly tested and validated by qRT‐PCR with multiple primer sets, and finally, miR‐676 was selected as a new potential candidate for a EV miR expression control. As shown in Figure 6A, miR‐676 indeed exhibited the most invariable expressions throughout all samples, which is comparable to U6. (Figure B and C). In fact, in regard to Ct values obtained by the Mann‐Whitney test between Sham and TAC EVs, miR‐676 expression (*P* = .52) was more consistent than U6 expression (*P* = .11). These data suggest that miR‐676 can be used as an internal control for EV miRs with high fidelity.

## 4 DISCUSSION

As EVs are highlighted as an important mediator of cell‐to‐cell communications during heart failure, it becomes essential to analyse the contents of cardiac EVs. In the present study, we isolated and characterized cardiac EVs from normal and failing mouse hearts. In the physical and molecular analyses, TAC EVs showed distinctive features in terms of density, size, protein expression patterns and miR contents as compared to those from Sham EVs (Figure 1). EVs are bound to cell surface proteins, extracellular matrix (ECM) molecules and components of the blood plasma through its surface molecules, and located in the niche of ECM or plasma.^25^ Several studies have characterized circulating EVs or miRs during cardiac diseases, but available information was highly limited since most of the studies were conducted using blood plasma.^26^, ^27^ Recently, Loyer X. et al investigated cardiac EVs in the model of myocardial infarction.^13^ In their report, they minced ventricles to obtain cardiac EVs, which might damage cardiac cells and increase potential contaminants from other intracellular components. In our study, on a contrary, Langendorff‐based EV isolation was employed to minimize damaging cells (Figure 1A). In addition, we have also compared our data with a previous study about the circulating miRs in a canine heart failure model.^27^ However, only thirteen miRs were confirmed to be identical out of fifty seven miRs reported in this paper (Table S1). This weak correlation suggests distinctive miR profiles of EVs in the ECM and plasma, which is probably due to different cellular origins of EVs. Plasma EVs could be released from the other organ or tissues, but EVs used for our study is strictly from cardiac tissues. In a similar context, we found the different size distribution and protein profiles between Sham and TAC EVs (Figure 1D‐F). In fact, our NTA data showed that the size of EVs in HF is larger than that of Sham. Previously, Minghua et al showed that size of exosomes were changed in remote ischaemic preconditioning‐mediated cardioprotection,^28^ which is correlated with our morphological observations. Taken together, these differences are also presumably related to the dynamic changes in the cellular composition of the heart under pathological conditions. In addition, we also observed the differences in the expression of exosomal markers. For example, CD63 and TSG101 were decreased in TAC EVs and these markers were previously reported to be involved in cardiac fibrosis and hypertrophy, respectively.^29^, ^30^ On the other hand, hnRNP A2B1 expression was increased and this was known to control the sorting of miRs into exosomes through specific binding motif.^31^ Although we particularly focused on miR profiles in EVs, this result also suggests a possibility that proteins in EVs could actively regulate cardiac intercellular communication.

From EV miR profiles, we have identified seven up‐regulated and three down‐regulated miRs in failing hearts compared to controls. Briefly, miR‐757, miR‐328, miR‐92b, miR‐139, miR‐665, miR‐378a and miR‐345 were up‐regulated, whereas, miR‐99b, miR‐124 and miR‐411 were down‐regulated during heart failure (Tables 1 and 2). Among the seven up‐regulated miRs, three miRs (miR‐92b, miR‐139 and miR‐378a) are known to be anti‐hypertrophic^31^, ^32^, ^33^, ^34^ and another three miRs (miR‐139, miR‐378a and miR‐345) are anti‐fibrotic^35^, ^36^, ^37^ (Table 5). These data indicate that up‐regulated miRs are likely involved in the cardioprotective function. Interestingly, one of down‐regulated miRs, miR‐124, is also known to induce cardiac hypertrophy,^38^ attenuate cardiac function and stimulate angiogenesis.^39^ Therefore, EV miRs released from failing hearts may play important roles in cardioprotection (Table 5). On the other hand, a negative‐inotropic miR, miR‐328, was found to be increased and an anti‐fibrotic miR, miR‐411, was decreased. In addition, we evaluated EV miR‐146a, a negative‐inotropic miR, that we previously reported,^10^ as a positive control and it was also significantly increased (Figure S1). Therefore, even though anti‐hypertrophic and anti‐fibrotic EV miRs are mainly elevated in TAC EVs, the negative‐inotropic and pro‐fibrotic miRs are also enriched. These ambivalent features of TAC EVs may result from diverse cellular origins of EVs, and EV miRs are meticulously balanced in normal heart. This finding is in line with the previous reports that EVs from cardiac stem cells are normally salubrious and the EVs from fibroblasts and endothelial cells are deleterious.^4^, ^6^, ^7^, ^8^, ^9^, ^10^, ^12^

**Table 5:** Previously described functions of differentially expressed miRs in TAC EV

Up‐regulated microRNAs				
Name	Contractility	Hypertrophy	Fibrosis	Reference
miR‐92b	Unknown	↓	‐	[33]
miR‐139	Unknown	↓	↓	[32, 36]
miR‐378a	Unknown	↓	↓	[34, 35]
miR‐345	Unknown	Unknown	↓	[37]
miR‐328	↓	↑	↑	[68, 69]
Down‐regulated microRNAs				
miR‐124	↓	↑		[38, 39]
miR‐411	Unknown	Unknown	↓	[70]

Furthermore, bioinformatics was applied as an alternative approach to predict the impact of EV miRs on cardiac function. First, we predicted the possible cardiac outcomes with previously reported targets for miRs identified in this study. Next, we performed literature studies and compared with previous results. In brief, molecular targets experimentally validated by both a reporter assay and Western blot analysis were initially selected based on the miR database (miRTarBase) and 52 targets that are not expressed in the heart were discarded. The remaining 77 putative targets of EV miRs are summarized in Table 3 (targets for up‐regulated miRs) and Table 4 (targets for down‐regulated miRs). Intriguingly, approximately 72% of targets anticipated to be up‐regulated were confirmed to be elevated indeed in diseased conditions by previous reports,^40^, ^41^, ^42^, ^43^, ^44^, ^45^, ^46^, ^47^, ^48^, ^49^, ^50^, ^51^ and 50% of targets expected to be down‐regulated were actually reduced.^52^, ^53^, ^54^, ^55^, ^56^, ^57^ This result proves that EV miRs from failing hearts substantially affect gene expressions in the heart.

Lastly, there have been controversial reports for the usage of U6 although it is the most frequently used endogenous reference control.^14^, ^15^, ^16^, ^17^, ^18^, ^19^ For example, U6 expression was not stable in tissues or plasma from patients with cancer.^14^, ^15^, ^17^ Therefore, we deliberated an extra assessment for the evaluation of EV miR expression. Initially, U6 was confirmed to be abundant and stable in our samples and used as a reference control in this study. To avoid potential critics for the usage of U6, we decided to identify alternative reference miR. Through the miRNA profile analysis, miR‐676 was appeared as a promising candidate due to its superior abundance and stability. Upon this result, additional normalization was performed using miR‐676 (Figure S2) and a significant correlation between U6 and miR‐676 was confirmed. Although miR‐676 is reported to be elevated in the liver in the response to a high‐fat diet,^58^ there are no reports relating to cardiac function. Therefore, miR‐676 may be a suitable reference in EV miR research although further validations are necessary in various cardiac diseases.

In summary, we isolated and characterized cardiac EVs in the context of miR contents using normal and failing hearts. EVs from both hearts exhibited substantial contrasts in physical and functional features. We also found that EVs from failing hearts contains both beneficial and detrimental miRs presumably because current EV miR profiles that we reported here were generated from mixed cellular origins in failing hearts, which is a limitation of this study.

Taken together, our study shows dynamic intercellular communications via EV miRs during heart failure and it will be critical to identify the origin of beneficial EVs for a therapeutic application to treat heart failure.

## CONFLICT OF INTEREST

The authors declare that they have no conflicts of interest.

## AUTHOR CONTRIBUTIONS

JGO, PL and DJ designed the research, performed the experiments, analysed the data and wrote the paper. REG contributed in performing EM microscopy. SS and CK served as scientific advisor. DJ revised a manuscript and data.

## Supporting information

Supplementary Material

## Data Availability

All data generated or analysed during this study are included in this published article (and its supplementary information files). Furthermore, the raw data sets generated during the current study are also available from the corresponding author on reasonable request.
