# Supplementary material for: Analysis of extracellular vesicle miRNA profiles in heart failure
Source: J Cell Mol Med. 2020 Jun 2;24(13):7214–27. doi: 10.1111/jcmm.15251 (PMC7339231; doi:10.1111/jcmm.15251)
Supplement: Supplementary file 1 — Supplementary Material [file JCMM-24-7214-s001.docx]

**Supplementary Data**

**Analysis of Extracellular Vesicle miRNA Profiles in Heart Failure**

Jae Gyun Oh, PhD^1,*^, Philyoung Lee^1,*^, Ronald E. Goldon, PhD^2^, Susmita Sahoo, PhD^1^, Changwon Kho, PhD^1^, and Dongtak Jeong, PhD^1,#^

^1^ Cardiovascular Research Center, Icahn School of Medicine at Mount Sinai, New York, New York, USA.

^2^Pathology Department, Icahn School of Medicine at Mount Sinai, New York, NY 10029

^#^Dongtak Jeong, PhD, Cardiovascular Research Center, Icahn School of Medicine at Mount Sinai (ISMMS), 1 Gustave L. Levy place, New York, New York, USA.

Tel.: +1-212-824-9007; Fax: +1-212-241-4080; E-mail: [dongtak.jeong@mssm.edu](mailto:Dongtak.jeong@mssm.edu).

*Both authors contributed equally to this manuscript.

**Supplementary Data**

***

**Supplementary figure 1**. The levels of miR-146a were quantified by qRT-PCR. The relative levels of miR-146a were normalized to the U6 snRNA. n=4. ***, p < 0.001 versus Sham Exo, as determined by one-way ANOVA. Data are presented as mean ± s.e.m.

n.s.

n.s.

n.s.

**

*

**

*

**

*

n.s.

A

n.s.

**

n.s.

n.s.

n.s.

**

*

B

**Supplementary figure 2. Validation of microRNA expression profiles by qRT-PCR.**

The levels of the upregulated (A) or downregulated (B) miRs were quantified by qRT-PCR. The relative levels of each microRNA were normalized to miR-676. n=4. *, p < 0.05, **, p < 0.01, ***, p < 0.001 versus Sham EV, as determined by one-way ANOVA. Data are presented as mean ± s.e.m.

B

A

**Supplementary figure 3. TAC-induced heart failure animal characterization.**

Two months after TAC operation, mice developed heart failure. (A, B) Echocardiographic parameters of heart failure animals. LV chamber dimensions and LV systolic function were calculated at 2 months of TAC mice and sham control. n = 6 (TAC) and n = 6 (Sham operated). Data are represented as mean ± SEM. * p < 0.05, *** p < 0.001 versus baseline (Student’s t-test). LVS, left ventricular systolic dimension

**Supplementary table 1.**

| **ID** | **Annotation** | **Log FC** | **P.Value** | **Sham EV 1** | **Sham EV 2** | **Sham EV 3** | **TAC EV 1** | **TAC EV 2** | **TAC EV 3** |
| --- | --- | --- | --- | --- | --- | --- | --- | --- | --- |
| 17511 | mmu-miR-713 | -6.0 | 1.2E-07 | 12.8 | 13.7 | 12.7 | 7.2 | 7.1 | 6.9 |
| 168810 | mmu-miR-5110 | -5.6 | 2.6E-04 | 14.4 | 10.7 | 12.3 | 7.2 | 7.0 | 6.4 |
| 148052 | mmu-miR-374c-3p | -5.3 | 5.5E-07 | 12.5 | 11.5 | 12.2 | 7.3 | 6.5 | 6.6 |
| 147203 | mmu-miR-302a-3p | -5.2 | 3.3E-08 | 13.6 | 14.4 | 14.0 | 8.6 | 8.8 | 8.8 |
| 148242 | mmu-miR-205-3p | -5.1 | 3.0E-07 | 10.2 | 11.3 | 10.7 | 5.8 | 5.5 | 5.7 |
| 42532 | mmu-miR-22-5p | -4.4 | 5.6E-05 | 12.1 | 9.9 | 10.6 | 6.8 | 6.4 | 6.4 |
| 168699 | mmu-miR-5627-5p | -4.3 | 1.4E-07 | 10.0 | 10.4 | 10.4 | 6.0 | 5.7 | 6.3 |
| 11184 | mmu-miR-99b-5p | -4.2 | 6.4E-06 | 12.3 | 11.0 | 11.1 | 7.3 | 7.2 | 7.2 |
| 148578 | mmu-miR-541-3p | -4.2 | 3.4E-04 | 14.1 | 11.2 | 12.4 | 8.3 | 8.5 | 8.3 |
| 33114 | mmu-miR-455-3p | -4.1 | 1.5E-08 | 11.2 | 11.4 | 11.4 | 7.1 | 7.4 | 7.2 |
| 17669 | mmu-miR-690 | -3.9 | 2.3E-06 | 9.7 | 10.8 | 10.1 | 6.2 | 6.0 | 6.5 |
| 19605 | SNORD6 | -3.9 | 7.5E-07 | 10.9 | 11.9 | 11.4 | 7.4 | 7.4 | 7.5 |
| 168715 | mmu-miR-5119 | -3.9 | 6.5E-05 | 10.2 | 8.2 | 9.6 | 5.5 | 5.4 | 5.4 |
| 11277 | mmu-miR-7a-1-3p | -3.5 | 2.3E-02 | 10.8 | 5.7 | 9.7 | 5.4 | 5.2 | 5.2 |
| 148415 | mmu-miR-668-5p | -3.4 | 4.0E-07 | 9.2 | 9.7 | 9.1 | 5.7 | 5.8 | 6.0 |
| 17422 | mmu-miR-695 | -3.4 | 7.1E-06 | 11.4 | 12.6 | 11.8 | 8.5 | 8.5 | 8.4 |
| 148281 | mmu-miR-467e-3p | -3.4 | 1.6E-05 | 10.0 | 8.8 | 9.7 | 6.4 | 6.0 | 5.9 |
| 28944 | mmu-miR-667-3p | -3.3 | 7.6E-08 | 10.7 | 10.5 | 10.9 | 7.3 | 7.5 | 7.4 |
| 17482 | mmu-miR-411-5p | -3.3 | 3.3E-05 | 7.9 | 9.3 | 8.0 | 5.2 | 5.1 | 5.1 |
| 148197 | mmu-miR-3081-5p | -3.1 | 7.0E-07 | 9.7 | 10.2 | 9.9 | 7.0 | 6.7 | 6.9 |
| 148087 | mmu-miR-669d-2-3p | -2.9 | 5.3E-03 | 9.9 | 6.7 | 9.3 | 5.7 | 5.6 | 5.7 |
| 148654 | mmu-miR-184-3p | -2.9 | 4.3E-04 | 10.0 | 8.1 | 8.5 | 6.2 | 6.1 | 5.7 |
| 168826 | mmu-miR-5624-5p | -2.7 | 2.0E-05 | 7.9 | 8.8 | 7.9 | 5.4 | 5.7 | 5.5 |
| 42471 | mmu-miR-290a-5p | -2.6 | 1.9E-06 | 12.4 | 12.5 | 12.7 | 10.3 | 9.7 | 9.9 |
| 42462 | mmu-miR-883a-5p | -2.4 | 1.3E-04 | 10.5 | 11.8 | 10.9 | 8.6 | 8.8 | 8.7 |
| 148646 | mmu-miR-467a-3p | -2.4 | 1.7E-06 | 8.6 | 8.2 | 8.7 | 6.2 | 6.1 | 6.1 |
| 147186 | mmu-miR-200b-3p | -2.4 | 2.1E-04 | 9.5 | 8.1 | 8.6 | 6.3 | 6.4 | 6.4 |
| 148017 | mmu-miR-743a-5p | -2.2 | 6.6E-03 | 9.2 | 6.6 | 8.3 | 5.7 | 5.7 | 5.8 |
| 14328 | mmu-miR-124-3p | -2.2 | 8.7E-04 | 8.2 | 9.8 | 8.4 | 6.6 | 6.5 | 6.6 |
| 148303 | mmu-miR-3106-5p | -2.1 | 3.5E-03 | 8.2 | 6.2 | 7.9 | 5.4 | 5.3 | 5.2 |
| 148344 | mmu-miR-669l-3p | -2.1 | 4.5E-04 | 10.5 | 9.2 | 10.5 | 7.8 | 8.1 | 8.1 |
| 145846 | mmu-let-7e-5p | -2.1 | 5.9E-06 | 9.3 | 9.8 | 9.4 | 7.4 | 7.5 | 7.4 |
| 146192 | mmu-miR-669m-3p | -1.8 | 7.1E-05 | 8.0 | 8.8 | 8.7 | 6.6 | 6.5 | 6.7 |
| 148485 | mghv-miR-M1-12-3p | -1.7 | 4.5E-06 | 9.5 | 9.4 | 9.7 | 7.8 | 7.9 | 7.8 |
| 148109 | mmu-miR-669a-3p/mmu-miR-669o-3p | -1.6 | 5.7E-05 | 7.0 | 6.7 | 7.4 | 5.4 | 5.4 | 5.4 |
| 148101 | mmu-miR-669d-2-3p/mmu-miR-669d-3p | -1.4 | 2.4E-03 | 10.7 | 9.5 | 10.6 | 8.7 | 9.0 | 8.9 |
| 148286 | mmu-miR-3066-3p | -1.4 | 1.4E-02 | 7.5 | 5.7 | 6.9 | 5.3 | 5.4 | 5.4 |
| 17278 | mmu-miR-719 | -1.3 | 4.1E-03 | 7.6 | 6.9 | 6.2 | 5.5 | 5.6 | 5.5 |
| 17290 | mghv-miR-M1-7-3p | -1.3 | 1.1E-04 | 8.2 | 8.7 | 8.7 | 7.2 | 7.1 | 7.3 |
| 46390 | mmu-miR-1192 | -1.3 | 1.1E-04 | 9.2 | 8.7 | 9.0 | 7.8 | 7.7 | 7.7 |
| 148559 | mmu-miR-411-3p | -1.2 | 5.2E-02 | 8.4 | 6.2 | 7.8 | 6.2 | 6.2 | 6.3 |
| 146147 | mmu-miR-1897-5p | -1.2 | 4.8E-03 | 12.0 | 11.1 | 10.9 | 10.0 | 10.2 | 10.2 |
| 32608 | mmu-miR-761 | -1.2 | 1.2E-03 | 6.6 | 7.3 | 6.5 | 5.6 | 5.6 | 5.6 |
| 46626 | mmu-miR-30c-2-3p | -1.2 | 3.7E-04 | 6.7 | 6.2 | 6.8 | 5.4 | 5.3 | 5.5 |
| 14289 | mmu-miR-540-3p | -1.2 | 6.4E-02 | 7.9 | 5.6 | 7.0 | 5.6 | 5.6 | 5.8 |
| 29852 | mmu-miR-9-3p | -1.1 | 2.6E-03 | 8.5 | 7.8 | 8.8 | 7.0 | 7.3 | 7.3 |
| 27565 | mmu-miR-423-5p | -1.1 | 6.9E-03 | 8.1 | 9.4 | 8.8 | 7.7 | 7.5 | 7.7 |
| 10928 | mmu-miR-125a-5p | -1.0 | 1.8E-03 | 7.6 | 8.4 | 8.2 | 6.9 | 7.0 | 7.1 |
| 42888 | mmu-miR-875-3p | -1.0 | 2.1E-01 | 10.9 | 7.8 | 10.2 | 8.7 | 8.6 | 8.7 |
| 148244 | mmu-miR-3098-3p | -1.0 | 1.8E-03 | 7.5 | 7.1 | 7.8 | 6.5 | 6.5 | 6.5 |
| 148226 | mmu-miR-467c-3p | -0.9 | 4.6E-02 | 7.1 | 5.4 | 6.4 | 5.4 | 5.3 | 5.4 |
| 42519 | mmu-miR-465c-5p | -0.9 | 1.9E-04 | 8.3 | 8.2 | 8.2 | 7.4 | 7.3 | 7.2 |
| 168662 | mmu-miR-5132-5p | -0.9 | 3.1E-01 | 9.5 | 5.8 | 7.7 | 6.5 | 6.9 | 6.9 |
| 42518 | mmu-miR-465b-5p | -0.9 | 7.8E-03 | 7.4 | 6.6 | 6.6 | 6.2 | 5.9 | 5.9 |
| 42605 | mmu-miR-503-3p | -0.9 | 4.2E-04 | 7.8 | 7.9 | 8.1 | 7.1 | 7.1 | 7.1 |
| 145661 | SNORD65 | -0.8 | 5.6E-02 | 7.1 | 8.5 | 7.1 | 6.8 | 6.8 | 6.6 |
| 145838 | mmu-miR-125b-1-3p | -0.8 | 6.3E-03 | 8.3 | 9.1 | 8.6 | 7.8 | 7.9 | 7.7 |
| 42502 | mmu-miR-204-3p | -0.8 | 2.0E-03 | 9.6 | 9.9 | 9.5 | 9.1 | 8.6 | 8.8 |
| 10306 | mmu-miR-146b-5p | -0.8 | 6.9E-04 | 7.0 | 7.1 | 7.2 | 6.2 | 6.4 | 6.2 |
| 42567 | mmu-miR-590-3p | -0.8 | 1.4E-03 | 7.1 | 7.5 | 7.1 | 6.3 | 6.6 | 6.4 |
| 14189 | mmu-miR-302b-5p | -0.8 | 1.2E-03 | 6.8 | 6.9 | 7.1 | 6.3 | 6.0 | 6.0 |
| 11218 | mmu-miR-294-3p | -0.8 | 2.8E-03 | 7.1 | 7.6 | 7.5 | 6.7 | 6.4 | 6.7 |
| 17431 | mghv-miR-M1-8-5p | -0.8 | 3.8E-03 | 8.1 | 8.7 | 8.5 | 7.8 | 7.5 | 7.6 |
| 11229 | mmu-miR-341-3p | -0.8 | 1.5E-01 | 9.1 | 7.1 | 8.8 | 7.5 | 7.6 | 7.5 |
| 11215 | mmu-miR-292a-3p | -0.8 | 7.6E-03 | 7.5 | 8.3 | 7.8 | 7.2 | 7.1 | 7.1 |
| 168968 | mmu-miR-147-3p | -0.7 | 1.0E-03 | 7.4 | 7.5 | 7.5 | 6.9 | 6.8 | 6.6 |
| 46210 | mmu-miR-1249-3p | -0.7 | 1.5E-02 | 7.6 | 8.4 | 7.8 | 7.2 | 7.2 | 7.3 |
| 17273 | mghv-miR-M1-6-3p | -0.7 | 2.4E-02 | 7.5 | 8.6 | 8.0 | 7.4 | 7.4 | 7.2 |
| 145889 | mmu-miR-196b-5p | -0.7 | 6.2E-03 | 6.8 | 7.4 | 7.2 | 6.5 | 6.5 | 6.2 |
| 28346 | mmu-miR-374b-3p | -0.7 | 4.1E-02 | 6.7 | 7.6 | 6.5 | 6.4 | 6.1 | 6.2 |
| 11227 | mmu-miR-329-3p | -0.7 | 2.3E-02 | 7.8 | 8.6 | 7.7 | 7.5 | 7.4 | 7.2 |
| 169248 | mmu-miR-5108 | -0.7 | 3.8E-03 | 6.6 | 7.1 | 6.9 | 6.0 | 6.1 | 6.3 |
| 168927 | mmu-miR-5136 | -0.7 | 4.7E-02 | 6.7 | 5.5 | 6.0 | 5.3 | 5.3 | 5.3 |
| 146039 | mmu-miR-669o-5p | -0.7 | 3.0E-02 | 7.8 | 8.8 | 8.0 | 7.7 | 7.5 | 7.3 |
| 148613 | mmu-miR-3110-5p | -0.7 | 2.3E-03 | 7.0 | 7.4 | 7.1 | 6.5 | 6.5 | 6.4 |
| 11226 | mmu-miR-325-5p | -0.7 | 1.9E-02 | 7.2 | 6.3 | 6.7 | 6.2 | 6.0 | 6.0 |
| 14288 | mmu-miR-503-5p | -0.7 | 1.3E-02 | 9.8 | 9.1 | 9.7 | 8.7 | 8.9 | 9.0 |
| 148523 | mghv-miR-M1-8-3p | -0.7 | 5.6E-03 | 8.0 | 8.6 | 8.3 | 7.8 | 7.5 | 7.6 |
| 145843 | mmu-miR-330-5p | -0.7 | 2.4E-02 | 7.7 | 8.5 | 7.7 | 7.4 | 7.3 | 7.3 |
| 4700 | mmu-miR-140-5p | -0.7 | 1.3E-02 | 7.2 | 7.8 | 7.4 | 6.9 | 6.9 | 6.6 |
| 42587 | mmu-miR-881-5p | -0.6 | 2.4E-01 | 7.6 | 5.7 | 7.5 | 6.3 | 6.1 | 6.4 |
| 42488 | mmu-miR-466h-5p | -0.6 | 1.3E-02 | 7.0 | 7.7 | 7.2 | 6.6 | 6.7 | 6.8 |
| 146030 | mmu-miR-2183 | -0.6 | 2.6E-03 | 8.0 | 8.2 | 8.0 | 7.5 | 7.4 | 7.4 |
| 11251 | mmu-miR-465a-5p | -0.6 | 2.1E-02 | 7.3 | 8.1 | 7.5 | 7.1 | 7.0 | 6.9 |
| 146087 | mmu-miR-1894-3p | -0.6 | 2.7E-02 | 10.1 | 10.8 | 10.5 | 9.7 | 10.1 | 9.8 |
| 46457 | mcmv-miR-M23-2-5p | -0.6 | 7.5E-03 | 6.4 | 6.8 | 6.5 | 6.2 | 5.9 | 5.8 |
| 27740 | mmu-miR-574-5p | -0.6 | 3.4E-02 | 7.1 | 8.1 | 7.6 | 7.0 | 6.9 | 6.9 |
| 17854 | mmu-miR-106b-3p | -0.6 | 5.5E-03 | 6.8 | 7.2 | 7.0 | 6.4 | 6.4 | 6.3 |
| 11208 | mmu-miR-207 | -0.6 | 3.2E-02 | 8.1 | 8.9 | 8.2 | 7.9 | 7.8 | 7.7 |
| 46481 | mcmv-miR-M44-1 | -0.6 | 3.3E-02 | 6.7 | 7.4 | 6.5 | 6.2 | 6.3 | 6.3 |
| 42507 | mmu-miR-202-5p | -0.6 | 9.1E-03 | 7.0 | 7.1 | 7.4 | 6.6 | 6.5 | 6.6 |
| 148107 | mmu-miR-3104-3p | -0.6 | 1.3E-02 | 7.4 | 8.0 | 7.7 | 7.1 | 7.1 | 7.3 |
| 42919 | mmu-miR-203-5p | -0.6 | 1.1E-01 | 6.6 | 7.3 | 6.0 | 6.1 | 6.1 | 5.9 |
| 42523 | mmu-miR-26b-3p | -0.6 | 1.4E-02 | 7.2 | 7.8 | 7.4 | 6.9 | 6.9 | 6.8 |
| 27536 | mmu-miR-190a-5p | -0.6 | 2.0E-02 | 7.1 | 7.7 | 7.3 | 6.9 | 6.9 | 6.7 |
| 11256 | mmu-miR-470-5p | -0.6 | 1.7E-01 | 7.6 | 9.1 | 8.3 | 7.9 | 7.7 | 7.7 |
| 42500 | mmu-miR-483-3p | -0.6 | 2.2E-02 | 7.3 | 8.0 | 7.6 | 7.1 | 7.0 | 7.1 |
| 148110 | mmu-miR-3075-3p | -0.6 | 5.9E-02 | 6.5 | 7.0 | 6.2 | 6.3 | 6.1 | 5.7 |
| 42913 | mmu-miR-345-3p | -0.5 | 1.1E-02 | 6.9 | 7.4 | 7.4 | 6.7 | 6.6 | 6.7 |
| 145641 | mmu-miR-369-5p | -0.5 | 1.2E-02 | 6.5 | 6.8 | 7.0 | 6.2 | 6.3 | 6.3 |
| 42636 | mmu-miR-28a-3p | -0.5 | 8.3E-03 | 6.5 | 6.8 | 6.7 | 6.0 | 6.2 | 6.3 |
| 46518 | mmu-miR-1198-5p | -0.5 | 6.5E-02 | 6.6 | 7.4 | 6.8 | 6.6 | 6.1 | 6.4 |
| 11182 | mmu-miR-98-5p | -0.5 | 5.6E-02 | 7.2 | 8.1 | 7.4 | 7.1 | 7.1 | 6.9 |
| 42528 | mmu-miR-296-3p | -0.5 | 2.7E-02 | 6.7 | 6.1 | 6.3 | 6.0 | 5.6 | 5.9 |
| 42626 | mmu-miR-30b-3p | -0.5 | 2.0E-02 | 7.0 | 7.5 | 7.3 | 6.8 | 6.8 | 6.6 |
| 46774 | mcmv-miR-m01-2-5p | -0.5 | 1.7E-02 | 8.4 | 8.2 | 7.8 | 7.6 | 7.6 | 7.6 |
| 17280 | mmu-miR-15b-5p | -0.5 | 2.6E-02 | 7.0 | 6.9 | 6.5 | 6.4 | 6.4 | 6.1 |
| 16528 | mmu-miR-706 | -0.5 | 2.4E-01 | 7.0 | 5.3 | 6.6 | 5.9 | 5.7 | 5.9 |
| 17851 | mmu-miR-200c-5p | -0.5 | 4.1E-02 | 6.4 | 6.8 | 7.1 | 6.4 | 6.3 | 6.1 |
| 46217 | mcmv-miR-m108-1-3p | -0.5 | 2.0E-02 | 6.6 | 7.2 | 7.0 | 6.5 | 6.4 | 6.3 |
| 46223 | mmu-miR-1306-3p | -0.5 | 3.4E-02 | 6.9 | 7.4 | 6.8 | 6.5 | 6.6 | 6.5 |
| 17312 | mmu-miR-592-5p | -0.5 | 1.3E-02 | 6.9 | 7.4 | 7.3 | 6.7 | 6.7 | 6.7 |
| 46489 | mmu-miR-669h-5p | -0.5 | 7.4E-03 | 6.6 | 6.6 | 6.6 | 5.9 | 6.2 | 6.1 |
| 10947 | mmu-miR-142a-3p | -0.5 | 1.8E-02 | 7.1 | 7.5 | 7.5 | 6.9 | 6.9 | 6.8 |
| 148200 | mmu-miR-3100-3p | -0.5 | 1.4E-02 | 10.1 | 9.7 | 10.0 | 9.3 | 9.3 | 9.6 |
| 146135 | mmu-miR-1968-5p | -0.5 | 2.1E-02 | 6.7 | 7.1 | 7.2 | 6.6 | 6.4 | 6.4 |
| 27720 | mmu-miR-15a-5p | -0.5 | 1.0E-02 | 6.9 | 7.3 | 7.1 | 6.6 | 6.6 | 6.6 |
| 14305 | mmu-miR-376b-5p | -0.5 | 1.4E-02 | 7.2 | 7.6 | 7.5 | 7.0 | 7.0 | 6.9 |
| 28966 | mmu-miR-574-3p | -0.5 | 2.9E-02 | 6.9 | 6.4 | 7.0 | 6.3 | 6.3 | 6.3 |
| 17810 | mmu-miR-29b-1-5p | -0.5 | 9.7E-02 | 7.0 | 7.7 | 6.8 | 6.7 | 6.9 | 6.5 |
| 4040 | mmu-miR-9-5p | -0.5 | 4.7E-02 | 6.9 | 7.6 | 7.2 | 6.9 | 6.8 | 6.6 |
| 148631 | mmu-miR-466j | -0.5 | 3.6E-02 | 6.9 | 7.5 | 7.2 | 6.7 | 6.7 | 6.7 |
| 11024 | mmu-miR-223-3p | -0.5 | 1.9E-02 | 6.9 | 7.3 | 7.2 | 6.5 | 6.6 | 6.8 |
| 146199 | mmu-miR-1961 | -0.5 | 6.6E-02 | 7.0 | 7.8 | 7.2 | 6.8 | 6.8 | 6.9 |
| 145666 | SNORD110 | -0.5 | 7.2E-03 | 7.9 | 7.9 | 7.9 | 7.5 | 7.4 | 7.4 |
| 146163 | mmu-miR-224-3p | -0.5 | 1.0E-01 | 7.1 | 6.1 | 6.7 | 6.1 | 6.2 | 6.3 |
| 10990 | mmu-miR-196a-5p | -0.5 | 4.2E-02 | 6.7 | 7.3 | 6.9 | 6.6 | 6.6 | 6.3 |
| 19585 | mmu-miR-148b-3p | -0.5 | 1.9E-02 | 7.0 | 7.4 | 7.2 | 6.8 | 6.6 | 6.7 |
| 42969 | mmu-miR-10b-3p | -0.5 | 8.4E-03 | 7.0 | 7.0 | 7.0 | 6.5 | 6.6 | 6.4 |
| 30831 | mmu-miR-804 | -0.5 | 2.8E-02 | 6.9 | 7.5 | 7.1 | 6.7 | 6.7 | 6.7 |
| 46615 | mcmv-miR-m01-2-3p | -0.5 | 6.8E-02 | 6.7 | 7.3 | 6.6 | 6.5 | 6.4 | 6.4 |
| 19603 | SNORD13 | -0.5 | 2.6E-02 | 6.7 | 7.2 | 7.0 | 6.5 | 6.5 | 6.6 |
| 19607 | SNORD15A | -0.5 | 4.7E-02 | 6.4 | 7.0 | 6.5 | 6.3 | 6.2 | 6.0 |
| 148187 | mmu-miR-410-5p | -0.5 | 2.3E-02 | 6.6 | 6.8 | 6.6 | 6.3 | 6.3 | 6.0 |
| 4390 | mmu-miR-7b-5p | -0.4 | 7.7E-02 | 7.3 | 8.0 | 7.3 | 7.2 | 7.1 | 6.9 |
| 148409 | mmu-miR-669k-5p | -0.4 | 4.2E-02 | 6.8 | 7.3 | 7.3 | 6.7 | 6.7 | 6.6 |
| 14301 | mmu-miR-361-5p | -0.4 | 3.7E-02 | 7.3 | 7.8 | 7.4 | 7.1 | 7.1 | 7.0 |
| 46243 | mmu-miR-1195 | -0.4 | 2.0E-02 | 6.4 | 6.7 | 6.6 | 6.2 | 6.1 | 6.2 |
| 17316 | mmu-miR-488-3p | -0.4 | 7.8E-02 | 6.6 | 7.1 | 7.1 | 6.7 | 6.6 | 6.2 |
| 4610 | mmu-miR-126a-3p | -0.4 | 3.6E-02 | 6.9 | 6.6 | 6.4 | 6.3 | 6.1 | 6.3 |
| 17304 | mmu-miR-683 | -0.4 | 4.9E-02 | 6.4 | 6.9 | 6.9 | 6.2 | 6.4 | 6.4 |
| 42460 | mmu-miR-223-5p | -0.4 | 1.9E-02 | 6.4 | 6.6 | 6.5 | 5.9 | 6.1 | 6.2 |
| 169347 | mmu-miR-5622-5p | -0.4 | 4.4E-02 | 6.6 | 6.3 | 6.8 | 6.2 | 6.1 | 6.2 |
| 46510 | mmu-miR-1188-5p | -0.4 | 8.7E-02 | 6.5 | 6.8 | 6.1 | 6.0 | 6.2 | 5.9 |
| 42898 | mmu-miR-124-5p | -0.4 | 3.6E-02 | 7.1 | 7.1 | 6.8 | 6.8 | 6.5 | 6.5 |
| 148081 | mmu-miR-3102-3p.2-3p | -0.4 | 1.2E-01 | 6.6 | 7.4 | 7.3 | 6.7 | 6.6 | 6.8 |
| 10946 | mmu-miR-141-3p | -0.4 | 2.9E-02 | 6.7 | 7.0 | 6.9 | 6.5 | 6.5 | 6.4 |
| 42615 | mmu-miR-135b-3p | -0.4 | 2.6E-02 | 6.5 | 6.7 | 6.5 | 6.1 | 6.3 | 6.2 |
| 11022 | mmu-miR-221-3p | -0.4 | 4.2E-02 | 6.8 | 7.3 | 7.0 | 6.7 | 6.7 | 6.5 |
| 33177 | mmu-miR-672-5p | -0.4 | 6.8E-02 | 6.8 | 7.3 | 6.8 | 6.6 | 6.6 | 6.5 |
| 27838 | mmu-miR-302d-3p | -0.4 | 7.0E-02 | 6.7 | 7.1 | 7.2 | 6.7 | 6.7 | 6.4 |
| 148055 | mmu-miR-3060-5p | -0.4 | 8.9E-02 | 6.3 | 6.7 | 6.9 | 6.2 | 6.3 | 6.3 |
| 42570 | mmu-miR-194-2-3p | -0.4 | 7.2E-02 | 7.3 | 6.8 | 7.2 | 6.7 | 6.8 | 6.6 |
| 148090 | mmu-miR-495-5p | -0.4 | 9.9E-02 | 7.4 | 6.7 | 7.2 | 6.6 | 6.9 | 6.7 |
| 6880 | mmu-miR-297a-5p | -0.4 | 4.7E-02 | 6.6 | 6.8 | 6.9 | 6.4 | 6.5 | 6.2 |
| 19011 | SNORD10 | -0.4 | 2.9E-02 | 6.3 | 6.6 | 6.6 | 6.1 | 6.2 | 6.1 |
| 148047 | mmu-miR-3058-3p | -0.4 | 9.8E-02 | 6.6 | 7.3 | 6.8 | 6.5 | 6.6 | 6.5 |
| 42810 | mmu-miR-149-5p | -0.4 | 1.1E-01 | 7.1 | 7.8 | 7.2 | 7.0 | 7.0 | 6.9 |
| 42771 | mmu-miR-877-3p | -0.4 | 7.5E-02 | 6.4 | 6.7 | 6.9 | 6.3 | 6.1 | 6.5 |
| 13485 | mmu-miR-10a-5p | -0.4 | 5.1E-02 | 6.8 | 6.8 | 6.4 | 6.3 | 6.4 | 6.2 |
| 42894 | mmu-miR-466e-5p | -0.4 | 2.4E-02 | 6.5 | 6.7 | 6.6 | 6.3 | 6.2 | 6.2 |
| 13147 | mmu-miR-96-5p | -0.4 | 5.4E-02 | 6.2 | 6.4 | 6.6 | 6.0 | 5.9 | 6.2 |
| 42674 | mmu-miR-431-3p | -0.4 | 7.2E-02 | 6.6 | 6.9 | 6.5 | 6.4 | 6.2 | 6.4 |
| 13140 | mmu-miR-138-5p | -0.4 | 8.1E-02 | 6.1 | 6.7 | 6.5 | 6.0 | 6.2 | 6.0 |
| 17904 | mmu-miR-185-3p | -0.4 | 2.8E-01 | 9.4 | 10.6 | 9.7 | 9.5 | 9.6 | 9.5 |
| 28480 | mmu-miR-504-5p | -0.3 | 2.3E-01 | 5.8 | 6.8 | 5.9 | 5.9 | 5.7 | 5.9 |
| 42665 | mmu-miR-543-5p | -0.3 | 2.7E-01 | 6.6 | 7.7 | 7.2 | 6.8 | 7.1 | 6.7 |
| 13784 | mmu-miR-547-3p | -0.3 | 2.4E-01 | 6.1 | 6.9 | 6.0 | 6.2 | 6.0 | 5.9 |
| 42733 | mmu-miR-702-3p | -0.3 | 1.2E-01 | 6.2 | 6.5 | 6.8 | 6.1 | 6.2 | 6.3 |
| 148521 | mmu-miR-466m-5p/mmu-miR-669m-5p | -0.3 | 1.2E-01 | 6.5 | 6.9 | 6.8 | 6.5 | 6.6 | 6.2 |
| 146013 | mmu-miR-1966-5p | -0.3 | 1.6E-01 | 6.5 | 7.0 | 6.6 | 6.2 | 6.7 | 6.3 |
| 17388 | mmu-miR-669a-5p/mmu-miR-669p-5p | -0.3 | 1.6E-01 | 6.5 | 7.2 | 6.8 | 6.6 | 6.6 | 6.4 |
| 168708 | mmu-miR-296-5p | -0.3 | 8.8E-02 | 6.7 | 7.0 | 7.1 | 6.7 | 6.5 | 6.6 |
| 42511 | mmu-miR-99a-3p | -0.3 | 2.1E-01 | 6.3 | 7.0 | 6.9 | 6.8 | 6.3 | 6.2 |
| 32731 | mmu-miR-190b-5p | -0.3 | 1.0E-01 | 6.2 | 6.6 | 6.5 | 6.3 | 6.1 | 6.0 |
| 46601 | mmu-miR-3059-5p | -0.3 | 5.4E-02 | 6.5 | 6.3 | 6.4 | 6.0 | 6.1 | 6.1 |
| 148134 | mmu-miR-3067-5p | -0.3 | 9.5E-02 | 6.4 | 6.8 | 6.7 | 6.4 | 6.4 | 6.3 |
| 148645 | mmu-miR-129-5p | -0.3 | 7.4E-02 | 6.3 | 6.4 | 6.7 | 6.2 | 6.2 | 6.2 |
| 146099 | mmu-miR-1950 | -0.3 | 1.2E-01 | 6.9 | 6.4 | 6.9 | 6.4 | 6.4 | 6.5 |
| 148636 | mmu-miR-466f | -0.3 | 1.1E-01 | 7.0 | 7.3 | 6.9 | 6.8 | 6.7 | 6.7 |
| 146193 | mmu-miR-1957a | -0.3 | 4.1E-01 | 7.7 | 6.4 | 6.5 | 6.4 | 6.7 | 6.6 |
| 11020 | mmu-miR-22-3p | -0.3 | 4.3E-01 | 7.7 | 6.4 | 7.7 | 6.9 | 7.2 | 6.8 |
| 42651 | mmu-miR-880-3p | -0.3 | 8.0E-02 | 6.0 | 6.3 | 6.3 | 5.9 | 5.9 | 5.9 |
| 42839 | mmu-miR-135a-5p | -0.3 | 9.7E-02 | 6.6 | 6.9 | 6.8 | 6.6 | 6.5 | 6.3 |
| 46381 | mmu-miR-1298-5p | -0.3 | 6.5E-02 | 6.5 | 6.7 | 6.6 | 6.4 | 6.3 | 6.3 |
| 148565 | mmu-miR-3113-3p | -0.3 | 1.6E-01 | 6.2 | 6.8 | 6.5 | 6.2 | 6.3 | 6.2 |
| 42494 | mmu-miR-712-3p | -0.3 | 1.8E-01 | 6.4 | 6.7 | 6.9 | 6.5 | 6.2 | 6.5 |
| 17752 | mmu-let-7f-5p | -0.3 | 1.4E-01 | 6.6 | 7.0 | 6.5 | 6.5 | 6.4 | 6.4 |
| 46453 | mmu-miR-466f-5p | -0.3 | 2.6E-01 | 6.8 | 7.6 | 6.9 | 7.0 | 6.7 | 6.8 |
| 146050 | mmu-miR-669n | -0.3 | 1.1E-01 | 7.1 | 7.3 | 6.9 | 6.9 | 6.9 | 6.7 |
| 11108 | mmu-miR-425-3p | -0.3 | 2.6E-01 | 9.0 | 9.8 | 9.2 | 9.0 | 9.1 | 9.1 |
| 28309 | mmu-miR-741-3p | -0.3 | 1.0E-01 | 6.4 | 6.7 | 6.6 | 6.3 | 6.2 | 6.3 |
| 146118 | mmu-miR-1894-5p | -0.3 | 1.8E-01 | 6.5 | 6.9 | 7.1 | 6.6 | 6.6 | 6.6 |
| 17427 | mmu-miR-200c-3p | -0.3 | 9.3E-02 | 6.7 | 6.6 | 6.6 | 6.5 | 6.2 | 6.4 |
| 11078 | mmu-miR-365-3p | -0.3 | 1.4E-01 | 7.1 | 7.5 | 7.5 | 7.1 | 7.1 | 7.1 |
| 42719 | mmu-miR-324-3p | -0.3 | 1.3E-01 | 6.5 | 6.7 | 6.9 | 6.4 | 6.4 | 6.4 |
| 17825 | mmu-miR-338-5p | -0.3 | 1.4E-01 | 6.7 | 6.6 | 6.5 | 6.4 | 6.5 | 6.1 |
| 148444 | mghv-miR-M1-2-5p | -0.3 | 2.8E-01 | 6.4 | 7.1 | 6.4 | 6.3 | 6.3 | 6.5 |
| 42945 | mmu-miR-297c-5p | -0.3 | 8.1E-02 | 6.2 | 6.2 | 6.2 | 6.0 | 5.9 | 5.9 |
| 147165 | mmu-let-7b-5p | -0.3 | 3.5E-01 | 6.7 | 7.7 | 6.9 | 6.8 | 6.9 | 6.8 |
| 42846 | mmu-miR-696 | -0.2 | 1.8E-01 | 6.1 | 6.6 | 6.2 | 6.2 | 6.0 | 6.0 |
| 146222 | mmu-miR-718 | -0.2 | 2.6E-01 | 6.1 | 6.5 | 6.7 | 6.4 | 6.1 | 6.0 |
| 148457 | mmu-miR-92b-5p | -0.2 | 2.8E-01 | 6.3 | 6.8 | 6.2 | 6.2 | 6.3 | 6.0 |
| 42592 | mmu-miR-338-3p | -0.2 | 1.4E-01 | 6.5 | 6.9 | 6.7 | 6.5 | 6.5 | 6.4 |
| 148143 | mmu-miR-466b-5p/mmu-miR-466o-5p | -0.2 | 1.8E-01 | 6.2 | 6.5 | 6.6 | 6.3 | 6.0 | 6.3 |
| 10925 | mmu-miR-10b-5p | -0.2 | 2.9E-01 | 6.5 | 6.8 | 6.5 | 6.4 | 6.7 | 6.1 |
| 46310 | mmu-miR-1187 | -0.2 | 1.8E-01 | 6.7 | 6.9 | 6.6 | 6.7 | 6.5 | 6.4 |
| 145705 | mmu-miR-431-5p | -0.2 | 3.5E-01 | 6.8 | 7.5 | 6.8 | 6.8 | 6.8 | 6.9 |
| 29562 | mmu-miR-199a-5p | -0.2 | 2.1E-01 | 6.5 | 6.7 | 6.8 | 6.4 | 6.3 | 6.6 |
| 17433 | mmu-miR-679-5p | -0.2 | 2.4E-01 | 6.5 | 6.9 | 6.8 | 6.3 | 6.6 | 6.6 |
| 42464 | mghv-miR-M1-2-3p | -0.2 | 2.5E-01 | 7.0 | 7.5 | 7.3 | 7.2 | 7.0 | 7.1 |
| 11074 | mmu-miR-34c-5p | -0.2 | 3.1E-01 | 6.5 | 7.0 | 6.5 | 6.6 | 6.5 | 6.3 |
| 11249 | mmu-miR-463-5p | -0.2 | 4.0E-01 | 6.1 | 6.8 | 6.3 | 6.4 | 6.4 | 5.9 |
| 10943 | mmu-miR-136-5p | -0.2 | 3.1E-01 | 6.4 | 6.6 | 6.2 | 6.2 | 6.4 | 6.0 |
| 42538 | mmu-miR-196a-2-3p | -0.2 | 5.7E-01 | 7.0 | 5.7 | 6.8 | 6.1 | 6.4 | 6.4 |
| 42456 | mmu-miR-598-3p | -0.2 | 2.9E-01 | 6.3 | 6.8 | 6.5 | 6.3 | 6.4 | 6.3 |
| 146026 | mmu-miR-1951 | -0.2 | 3.0E-01 | 6.2 | 6.5 | 6.0 | 6.1 | 6.1 | 6.1 |
| 146195 | mmu-miR-2139 | -0.2 | 2.8E-01 | 6.7 | 6.4 | 6.8 | 6.4 | 6.4 | 6.5 |
| 148267 | mmu-miR-3082-5p | -0.2 | 3.0E-01 | 6.8 | 7.3 | 7.0 | 6.9 | 6.8 | 6.8 |
| 30768 | mmu-miR-674-5p | -0.2 | 2.6E-01 | 6.4 | 6.8 | 6.5 | 6.4 | 6.4 | 6.4 |
| 9938 | mmu-let-7i-5p | -0.2 | 2.8E-01 | 6.3 | 6.7 | 6.4 | 6.3 | 6.3 | 6.3 |
| 46917 | mmu-miR-205-5p | -0.2 | 6.2E-01 | 7.2 | 7.9 | 6.6 | 7.1 | 7.0 | 6.9 |
| 145827 | mmu-miR-200a-5p | -0.2 | 3.9E-01 | 6.3 | 6.5 | 6.2 | 6.0 | 6.5 | 6.1 |
| 148476 | mmu-miR-3552 | -0.2 | 3.5E-01 | 6.4 | 6.6 | 6.8 | 6.4 | 6.4 | 6.6 |
| 14313 | mmu-miR-499-5p | -0.2 | 4.4E-01 | 6.6 | 7.0 | 6.5 | 6.5 | 6.8 | 6.4 |
| 17378 | mmu-miR-698-3p | -0.2 | 4.4E-01 | 6.6 | 6.8 | 6.8 | 6.6 | 6.9 | 6.3 |
| 148470 | mmu-miR-1264-3p | -0.1 | 5.0E-01 | 7.2 | 6.6 | 7.3 | 6.9 | 6.9 | 6.8 |
| 146128 | mmu-miR-1982-3p | -0.1 | 4.0E-01 | 6.4 | 6.6 | 6.5 | 6.6 | 6.3 | 6.2 |
| 19601 | mmu-miR-211-5p | -0.1 | 5.3E-01 | 6.0 | 6.7 | 6.2 | 6.3 | 6.1 | 6.0 |
| 27558 | mmu-miR-155-5p | -0.1 | 4.3E-01 | 6.5 | 6.5 | 6.7 | 6.7 | 6.3 | 6.3 |
| 168807 | mmu-miR-3473c | -0.1 | 6.1E-01 | 6.2 | 6.4 | 7.0 | 6.4 | 6.1 | 6.7 |
| 17853 | mmu-miR-30d-3p | -0.1 | 3.3E-01 | 6.0 | 6.2 | 6.1 | 5.9 | 6.0 | 6.0 |
| 42929 | mmu-miR-25-5p | -0.1 | 6.2E-01 | 8.5 | 7.5 | 8.1 | 7.9 | 7.9 | 7.9 |
| 145820 | mmu-let-7c-5p | -0.1 | 5.7E-01 | 6.5 | 7.2 | 6.5 | 6.6 | 6.5 | 6.7 |
| 42865 | mmu-miR-181a-5p | -0.1 | 4.2E-01 | 6.1 | 6.4 | 6.3 | 6.1 | 6.2 | 6.1 |
| 148480 | mmu-miR-494-5p | -0.1 | 7.0E-01 | 6.1 | 6.8 | 5.8 | 6.0 | 6.5 | 6.0 |
| 148236 | mghv-miR-M1-15 | -0.1 | 5.5E-01 | 6.2 | 6.5 | 6.5 | 6.2 | 6.1 | 6.5 |
| 42899 | mmu-miR-377-5p | -0.1 | 4.6E-01 | 6.7 | 6.9 | 6.7 | 6.7 | 6.7 | 6.7 |
| 147283 | mmu-miR-137-5p | -0.1 | 6.6E-01 | 6.3 | 6.9 | 6.2 | 6.3 | 6.4 | 6.4 |
| 19013 | SNORD14B | -0.1 | 6.3E-01 | 6.2 | 6.6 | 6.7 | 6.2 | 6.6 | 6.4 |
| 42579 | mmu-miR-193a-5p | -0.1 | 5.8E-01 | 6.1 | 6.4 | 6.3 | 6.1 | 6.0 | 6.4 |
| 148035 | mmu-miR-3084-5p | -0.1 | 5.7E-01 | 6.5 | 6.9 | 6.9 | 6.7 | 6.6 | 6.7 |
| 46297 | mmu-miR-3085-3p | -0.1 | 6.8E-01 | 5.8 | 6.3 | 6.5 | 6.2 | 6.1 | 6.2 |
| 42687 | mmu-miR-883b-5p | -0.1 | 6.1E-01 | 8.5 | 8.7 | 8.7 | 8.4 | 8.7 | 8.5 |
| 17517 | mmu-miR-688 | -0.1 | 6.7E-01 | 7.5 | 7.1 | 7.0 | 7.2 | 7.2 | 7.1 |
| 30033 | mmu-miR-877-5p | 0.0 | 7.4E-01 | 7.7 | 8.1 | 7.9 | 7.8 | 7.8 | 7.9 |
| 29490 | mmu-miR-7a-5p | 0.0 | 7.6E-01 | 6.8 | 7.1 | 6.8 | 6.9 | 6.9 | 6.8 |
| 11005 | mmu-miR-204-5p | 0.0 | 7.4E-01 | 6.4 | 6.7 | 6.5 | 6.5 | 6.5 | 6.5 |
| 14271 | mmu-miR-539-5p | 0.0 | 8.6E-01 | 6.3 | 6.9 | 6.3 | 6.2 | 6.7 | 6.5 |
| 14290 | mmu-miR-541-5p | 0.0 | 8.4E-01 | 6.1 | 6.5 | 6.5 | 6.5 | 6.2 | 6.4 |
| 46346 | mmu-miR-669e-5p | 0.0 | 8.2E-01 | 6.6 | 6.7 | 6.5 | 6.7 | 6.5 | 6.4 |
| 32946 | mmu-miR-486a-5p/mmu-miR-486b-5p | 0.0 | 8.4E-01 | 6.5 | 6.8 | 6.6 | 6.5 | 6.7 | 6.6 |
| 148099 | mmu-miR-344h-3p | 0.0 | 8.9E-01 | 8.6 | 8.6 | 8.8 | 8.7 | 8.7 | 8.6 |
| 146221 | mmu-miR-669c-5p | 0.0 | 9.4E-01 | 7.3 | 7.0 | 7.5 | 7.2 | 7.3 | 7.3 |
| 27571 | mmu-miR-292a-5p | 0.0 | 9.7E-01 | 6.2 | 6.8 | 6.3 | 6.5 | 6.4 | 6.4 |
| 146156 | mmu-miR-1960 | 0.0 | 1.0E+00 | 7.2 | 7.7 | 7.2 | 7.4 | 7.4 | 7.4 |
| 146002 | mmu-miR-669l-5p | 0.0 | 9.5E-01 | 6.0 | 6.1 | 6.4 | 6.2 | 6.2 | 6.3 |
| 146008 | mmu-miR-26b-5p | 0.0 | 9.5E-01 | 6.3 | 6.7 | 6.5 | 6.5 | 6.4 | 6.6 |
| 42569 | mmu-miR-871-5p | 0.0 | 9.4E-01 | 6.3 | 6.7 | 6.1 | 6.3 | 6.7 | 6.1 |
| 169373 | mmu-miR-5626-5p | 0.0 | 8.9E-01 | 7.3 | 7.4 | 7.4 | 7.4 | 7.3 | 7.4 |
| 11254 | mmu-miR-468-3p | 0.0 | 8.7E-01 | 6.1 | 6.0 | 6.2 | 6.2 | 6.1 | 6.1 |
| 33163 | mmu-miR-676-3p | 0.0 | 8.5E-01 | 6.2 | 6.2 | 6.3 | 6.4 | 6.1 | 6.3 |
| 148570 | mmu-miR-466n-5p | 0.0 | 8.1E-01 | 6.2 | 6.4 | 6.1 | 6.3 | 6.3 | 6.2 |
| 148432 | mghv-miR-M1-10-3p | 0.0 | 8.3E-01 | 6.7 | 6.4 | 6.5 | 6.5 | 6.5 | 6.8 |
| 42594 | mmu-miR-453 | 0.0 | 8.6E-01 | 6.2 | 6.5 | 6.2 | 6.7 | 6.4 | 5.9 |
| 148218 | mghv-miR-M1-11-3p | 0.0 | 7.7E-01 | 7.1 | 6.9 | 7.0 | 7.1 | 7.1 | 7.0 |
| 169268 | mmu-miR-5112 | 0.0 | 8.9E-01 | 7.9 | 6.9 | 7.8 | 7.9 | 7.3 | 7.5 |
| 146175 | mmu-miR-1896 | 0.1 | 7.5E-01 | 6.3 | 6.8 | 6.5 | 6.6 | 6.7 | 6.5 |
| 17465 | mmu-miR-678 | 0.1 | 8.5E-01 | 7.0 | 8.1 | 7.3 | 7.4 | 7.5 | 7.4 |
| 42703 | mmu-miR-490-3p | 0.1 | 9.2E-01 | 8.2 | 5.7 | 7.2 | 7.1 | 7.1 | 7.1 |
| 168596 | mmu-miR-5620-3p | 0.1 | 6.9E-01 | 6.2 | 6.4 | 6.4 | 6.4 | 6.3 | 6.5 |
| 146176 | mmu-miR-1971 | 0.1 | 8.1E-01 | 10.3 | 11.2 | 10.8 | 10.3 | 11.0 | 11.1 |
| 146125 | mmu-miR-1903 | 0.1 | 8.2E-01 | 7.1 | 5.8 | 6.3 | 6.7 | 6.3 | 6.4 |
| 168689 | mmu-miR-361-3p | 0.1 | 5.8E-01 | 7.3 | 7.0 | 7.2 | 7.2 | 7.3 | 7.2 |
| 148614 | mmu-miR-7a-2-3p | 0.1 | 7.8E-01 | 7.5 | 8.7 | 8.3 | 8.2 | 8.3 | 8.2 |
| 11221 | mmu-miR-300-3p | 0.1 | 8.4E-01 | 6.8 | 8.7 | 6.8 | 7.5 | 7.5 | 7.6 |
| 42927 | mmu-miR-673-3p | 0.1 | 5.3E-01 | 7.7 | 8.4 | 7.9 | 8.1 | 8.2 | 8.1 |
| 17621 | mmu-miR-701-5p | 0.1 | 4.0E-01 | 6.3 | 6.0 | 6.4 | 6.4 | 6.3 | 6.4 |
| 145753 | mmu-miR-484 | 0.1 | 3.8E-01 | 6.5 | 6.7 | 6.8 | 6.6 | 6.9 | 6.8 |
| 148433 | mmu-miR-466i-5p | 0.2 | 3.5E-01 | 6.4 | 6.7 | 6.7 | 6.7 | 6.7 | 6.8 |
| 29575 | mmu-miR-32-3p | 0.2 | 2.5E-01 | 6.2 | 6.3 | 6.4 | 6.4 | 6.4 | 6.5 |
| 148059 | mmu-miR-493-5p | 0.2 | 3.0E-01 | 7.1 | 6.6 | 7.1 | 7.3 | 6.9 | 7.1 |
| 148446 | mmu-miR-346-3p | 0.2 | 3.3E-01 | 9.1 | 10.0 | 9.6 | 9.8 | 9.8 | 9.9 |
| 148146 | mmu-miR-3076-3p | 0.2 | 1.4E-01 | 6.3 | 6.3 | 6.6 | 6.5 | 6.7 | 6.7 |
| 11245 | mmu-miR-433-5p | 0.2 | 4.9E-01 | 10.9 | 9.5 | 10.3 | 10.5 | 10.2 | 10.8 |
| 148689 | mmu-miR-3099-5p | 0.3 | 1.5E-01 | 7.1 | 6.9 | 7.3 | 7.3 | 7.4 | 7.3 |
| 30787 | mmu-miR-125b-5p | 0.3 | 1.4E-01 | 6.6 | 7.0 | 6.8 | 7.0 | 7.1 | 7.1 |
| 168794 | mmu-miR-5107-5p | 0.3 | 3.9E-01 | 6.9 | 8.0 | 7.0 | 7.7 | 7.6 | 7.5 |
| 17898 | mmu-miR-99b-3p | 0.3 | 1.8E-01 | 7.2 | 6.6 | 6.9 | 7.2 | 7.1 | 7.1 |
| 148558 | mmu-miR-3064-5p | 0.3 | 6.4E-02 | 6.4 | 6.6 | 6.6 | 6.8 | 6.8 | 6.8 |
| 42922 | mmu-miR-450a-2-3p | 0.3 | 4.4E-01 | 8.1 | 6.7 | 7.7 | 7.9 | 7.8 | 7.7 |
| 10936 | mmu-miR-130b-3p | 0.3 | 1.1E-01 | 6.3 | 6.7 | 6.7 | 6.9 | 6.8 | 6.9 |
| 42879 | mmu-miR-92a-2-5p | 0.3 | 5.6E-02 | 7.8 | 8.1 | 8.0 | 8.2 | 8.4 | 8.2 |
| 146088 | mmu-miR-1983 | 0.3 | 9.1E-02 | 7.5 | 7.3 | 7.8 | 7.9 | 7.9 | 7.7 |
| 148651 | mmu-miR-3072-3p | 0.3 | 9.5E-02 | 6.4 | 6.3 | 6.8 | 6.9 | 6.7 | 6.8 |
| 17896 | mmu-miR-21a-3p | 0.3 | 1.4E-01 | 10.6 | 11.3 | 11.1 | 11.5 | 11.4 | 11.2 |
| 17352 | mghv-miR-M1-5-5p | 0.3 | 3.4E-02 | 8.0 | 8.2 | 8.1 | 8.4 | 8.4 | 8.5 |
| 148362 | mmu-miR-592-3p | 0.4 | 5.6E-01 | 9.7 | 7.2 | 9.5 | 9.1 | 9.3 | 9.1 |
| 17835 | mmu-miR-450a-5p | 0.4 | 4.6E-02 | 6.7 | 7.1 | 7.1 | 7.4 | 7.3 | 7.4 |
| 14272 | mmu-miR-542-3p | 0.4 | 1.9E-01 | 7.1 | 8.2 | 7.7 | 7.9 | 8.2 | 8.0 |
| 148653 | mmu-miR-3474 | 0.4 | 1.7E-02 | 8.7 | 8.8 | 8.8 | 9.1 | 9.2 | 9.2 |
| 169153 | mmu-miR-5116 | 0.4 | 1.4E-02 | 9.2 | 9.1 | 9.2 | 9.7 | 9.5 | 9.6 |
| 145977 | mmu-miR-1247-5p | 0.4 | 2.6E-02 | 6.7 | 6.9 | 6.6 | 7.1 | 7.1 | 7.3 |
| 146164 | mmu-miR-1958 | 0.4 | 3.0E-02 | 6.9 | 6.6 | 6.5 | 7.0 | 7.1 | 7.2 |
| 28547 | mmu-miR-675-5p | 0.4 | 3.6E-01 | 7.8 | 9.7 | 8.4 | 9.0 | 9.1 | 9.1 |
| 145643 | mmu-miR-382-5p | 0.4 | 5.8E-02 | 6.2 | 6.6 | 5.9 | 6.6 | 6.8 | 6.7 |
| 11007 | mmu-miR-206-3p | 0.4 | 1.2E-02 | 6.4 | 6.5 | 6.3 | 6.8 | 6.9 | 6.8 |
| 148230 | mmu-miR-450a-1-3p | 0.4 | 3.6E-01 | 8.6 | 6.7 | 8.0 | 8.5 | 8.1 | 8.0 |
| 42509 | mmu-miR-219a-5p | 0.4 | 1.2E-02 | 6.1 | 6.2 | 6.0 | 6.5 | 6.6 | 6.5 |
| 19606 | SNORD12 | 0.5 | 1.5E-02 | 6.2 | 6.3 | 6.5 | 6.8 | 6.7 | 6.9 |
| 10923 | mmu-miR-107-3p | 0.5 | 4.8E-02 | 7.9 | 8.6 | 8.3 | 8.8 | 8.7 | 8.6 |
| 29872 | mmu-miR-340-5p | 0.5 | 1.7E-02 | 6.3 | 6.2 | 6.6 | 6.9 | 6.7 | 6.9 |
| 145745 | mmu-miR-335-3p | 0.5 | 1.1E-02 | 9.1 | 9.1 | 8.8 | 9.5 | 9.5 | 9.4 |
| 42851 | mmu-miR-105 | 0.5 | 2.2E-02 | 6.1 | 6.4 | 6.1 | 6.5 | 6.8 | 6.8 |
| 17506 | mmu-miR-24-3p | 0.5 | 8.3E-02 | 6.4 | 7.3 | 6.5 | 7.2 | 7.4 | 7.2 |
| 146160 | mmu-miR-133b-3p | 0.5 | 1.5E-02 | 7.0 | 7.5 | 7.3 | 7.9 | 7.8 | 7.7 |
| 23524 | mmu-miR-465a-3p/mmu-miR-465b-3p/mmu-miR-465c-3p | 0.5 | 6.7E-03 | 6.3 | 6.4 | 6.3 | 7.0 | 6.7 | 6.9 |
| 42706 | mmu-miR-325-3p | 0.5 | 2.0E-02 | 7.7 | 7.3 | 7.8 | 8.0 | 8.3 | 8.0 |
| 10919 | mmu-miR-103-3p | 0.6 | 1.5E-02 | 8.3 | 8.8 | 8.4 | 9.2 | 9.1 | 9.0 |
| 146097 | mmu-miR-1934-5p | 0.6 | 7.6E-03 | 8.1 | 8.3 | 8.4 | 8.7 | 8.7 | 9.0 |
| 146137 | mmu-miR-133a-3p | 0.6 | 3.5E-02 | 6.4 | 7.1 | 7.0 | 7.5 | 7.3 | 7.4 |
| 168706 | mmu-miR-5129-5p | 0.6 | 2.3E-02 | 8.0 | 7.6 | 7.8 | 8.7 | 8.1 | 8.3 |
| 17610 | mmu-miR-677-5p | 0.6 | 4.1E-03 | 6.7 | 6.7 | 6.8 | 7.2 | 7.5 | 7.3 |
| 14316 | mmu-miR-664-3p | 0.6 | 5.0E-02 | 6.9 | 6.0 | 6.7 | 7.1 | 7.1 | 7.1 |
| 10916 | mmu-miR-1a-3p | 0.6 | 8.4E-02 | 6.3 | 7.1 | 6.4 | 7.7 | 6.7 | 7.3 |
| 146004 | mmu-miR-2136 | 0.6 | 5.1E-02 | 6.9 | 7.6 | 6.6 | 7.7 | 7.7 | 7.6 |
| 148609 | mmu-miR-487b-5p | 0.6 | 7.5E-03 | 6.3 | 5.9 | 6.0 | 6.5 | 6.8 | 6.8 |
| 148690 | mmu-miR-466d-5p | 0.6 | 3.4E-03 | 5.9 | 6.0 | 5.9 | 6.7 | 6.7 | 6.4 |
| 168752 | mmu-miR-5627-3p | 0.6 | 2.3E-03 | 6.7 | 6.7 | 6.9 | 7.4 | 7.3 | 7.5 |
| 148020 | mmu-miR-3078-3p | 0.7 | 2.8E-02 | 7.9 | 7.4 | 8.3 | 8.4 | 8.5 | 8.6 |
| 14285 | mmu-miR-487b-3p | 0.7 | 2.8E-02 | 7.3 | 6.4 | 6.5 | 7.5 | 7.4 | 7.3 |
| 17291 | mghv-miR-M1-4-5p | 0.7 | 7.7E-04 | 9.0 | 9.0 | 9.0 | 9.8 | 9.7 | 9.7 |
| 148051 | mmu-miR-770-3p | 0.8 | 2.3E-03 | 6.7 | 6.7 | 7.0 | 7.3 | 7.7 | 7.6 |
| 42452 | mmu-miR-141-5p | 0.8 | 2.2E-03 | 6.0 | 5.9 | 6.0 | 6.5 | 7.0 | 6.8 |
| 146106 | mmu-miR-1931 | 0.8 | 1.2E-03 | 6.3 | 6.2 | 6.6 | 7.2 | 7.1 | 7.2 |
| 146057 | mmu-miR-1967 | 0.8 | 4.8E-04 | 6.7 | 6.4 | 6.6 | 7.4 | 7.4 | 7.4 |
| 168977 | mmu-miR-5128 | 0.8 | 5.7E-04 | 6.1 | 5.9 | 6.1 | 6.7 | 6.9 | 6.9 |
| 42868 | mmu-miR-762 | 0.8 | 7.3E-04 | 8.6 | 8.8 | 8.7 | 9.7 | 9.4 | 9.4 |
| 145633 | mmu-let-7d-3p | 0.8 | 1.0E-03 | 8.2 | 8.2 | 8.3 | 8.9 | 9.3 | 9.0 |
| 148097 | mmu-miR-329-5p | 0.8 | 7.2E-04 | 6.1 | 6.2 | 6.2 | 6.8 | 7.2 | 7.1 |
| 28019 | mmu-miR-10a-3p | 0.8 | 9.5E-04 | 7.5 | 7.2 | 7.3 | 8.3 | 8.1 | 8.0 |
| 148632 | mmu-miR-2861 | 0.8 | 6.5E-04 | 8.1 | 7.8 | 8.1 | 8.8 | 8.8 | 8.9 |
| 148199 | mmu-miR-3102-3p | 0.8 | 3.8E-03 | 6.4 | 5.7 | 5.9 | 6.9 | 6.8 | 6.9 |
| 168607 | mmu-miR-299a-5p | 0.8 | 3.1E-03 | 9.0 | 8.5 | 9.0 | 9.6 | 9.6 | 9.9 |
| 19600 | mmu-miR-17-3p | 0.8 | 1.0E-02 | 7.4 | 6.4 | 6.9 | 7.9 | 7.6 | 7.7 |
| 42490 | mmu-miR-505-5p | 0.9 | 8.1E-04 | 7.3 | 6.9 | 7.1 | 8.1 | 7.9 | 8.0 |
| 148325 | mmu-miR-1981-3p | 0.9 | 6.9E-02 | 7.8 | 6.2 | 7.6 | 7.9 | 8.1 | 8.2 |
| 148484 | mmu-miR-3084-3p | 0.9 | 2.1E-03 | 6.3 | 6.3 | 6.4 | 6.9 | 7.5 | 7.4 |
| 17953 | mmu-miR-183-3p | 0.9 | 5.1E-04 | 8.8 | 8.8 | 8.6 | 9.8 | 9.5 | 9.6 |
| 11235 | mmu-miR-351-5p | 0.9 | 2.0E-02 | 8.7 | 7.6 | 8.6 | 9.1 | 9.3 | 9.1 |
| 42609 | mmu-miR-135a-1-3p | 0.9 | 8.9E-04 | 6.2 | 6.3 | 6.1 | 7.2 | 6.8 | 7.3 |
| 46251 | mmu-miR-1193-3p | 0.9 | 1.5E-04 | 6.4 | 6.3 | 6.5 | 7.3 | 7.3 | 7.4 |
| 11210 | mmu-miR-215-5p | 0.9 | 6.7E-04 | 6.1 | 5.7 | 6.0 | 6.8 | 6.8 | 6.9 |
| 42585 | mmu-miR-297a-3p/mmu-miR-297b-3p/mmu-miR-297c-3p | 0.9 | 1.3E-03 | 6.5 | 6.0 | 6.2 | 7.0 | 7.4 | 7.1 |
| 148468 | mmu-miR-677-3p | 1.0 | 2.5E-03 | 8.8 | 9.0 | 9.3 | 9.7 | 10.3 | 9.9 |
| 148437 | mmu-miR-3086-3p | 1.0 | 5.6E-02 | 7.0 | 5.3 | 5.5 | 7.0 | 6.7 | 7.1 |
| 169111 | mmu-miR-5616-3p | 1.0 | 5.4E-04 | 8.3 | 8.3 | 7.8 | 9.1 | 9.1 | 9.2 |
| 145663 | SNORD68 | 1.0 | 1.1E-02 | 6.9 | 5.7 | 6.5 | 7.4 | 7.4 | 7.3 |
| 17537 | mghv-miR-M1-3-3p | 1.0 | 1.5E-04 | 6.7 | 6.5 | 6.8 | 7.6 | 7.7 | 7.7 |
| 19008 | SNORD2 | 1.0 | 3.6E-02 | 8.0 | 6.3 | 7.1 | 8.3 | 8.1 | 8.0 |
| 31388 | mmu-miR-291a-5p | 1.0 | 2.4E-04 | 7.6 | 7.2 | 7.6 | 8.5 | 8.4 | 8.5 |
| 148068 | mmu-miR-758-5p | 1.0 | 2.5E-04 | 5.6 | 5.8 | 5.7 | 6.7 | 6.5 | 6.9 |
| 27672 | mmu-miR-615-3p | 1.0 | 2.2E-04 | 6.5 | 6.7 | 6.6 | 7.5 | 7.8 | 7.5 |
| 42738 | mmu-miR-340-3p | 1.0 | 2.7E-04 | 6.0 | 5.7 | 5.6 | 6.8 | 6.7 | 6.9 |
| 28505 | mmu-miR-676-5p | 1.0 | 9.4E-05 | 5.6 | 5.6 | 5.7 | 6.5 | 6.7 | 6.7 |
| 146170 | mmu-miR-1902 | 1.0 | 5.6E-05 | 6.3 | 6.3 | 6.2 | 7.3 | 7.2 | 7.3 |
| 42445 | mmu-miR-693-5p | 1.1 | 1.2E-04 | 8.3 | 8.5 | 8.6 | 9.4 | 9.6 | 9.6 |
| 168835 | mmu-miR-5621-5p | 1.1 | 7.6E-05 | 5.4 | 5.5 | 5.5 | 6.6 | 6.4 | 6.6 |
| 148158 | mghv-miR-M1-5-3p | 1.1 | 2.0E-02 | 7.3 | 5.8 | 6.4 | 7.5 | 7.6 | 7.6 |
| 146054 | mmu-miR-1952 | 1.1 | 2.3E-04 | 8.7 | 9.0 | 9.0 | 9.8 | 10.1 | 10.0 |
| 148608 | mmu-miR-551b-5p | 1.1 | 7.2E-05 | 5.7 | 5.5 | 5.6 | 6.7 | 6.6 | 6.8 |
| 42606 | mmu-miR-330-3p | 1.1 | 4.7E-04 | 5.9 | 5.6 | 6.0 | 7.0 | 6.7 | 7.1 |
| 11014 | mmu-miR-214-3p | 1.1 | 4.5E-04 | 6.0 | 5.4 | 6.0 | 6.8 | 6.9 | 7.0 |
| 145994 | mmu-miR-1900 | 1.1 | 2.4E-02 | 10.0 | 11.0 | 10.6 | 10.9 | 11.9 | 12.2 |
| 42752 | mmu-miR-872-3p | 1.1 | 9.8E-05 | 7.6 | 8.0 | 7.7 | 9.0 | 8.9 | 8.9 |
| 146055 | mmu-miR-1954 | 1.2 | 8.7E-05 | 6.9 | 7.2 | 7.1 | 8.3 | 8.2 | 8.2 |
| 148309 | mmu-miR-3068-3p | 1.2 | 1.3E-01 | 5.7 | 8.7 | 7.4 | 8.3 | 8.5 | 8.6 |
| 146082 | mmu-miR-1956 | 1.2 | 3.9E-05 | 7.2 | 7.1 | 7.2 | 8.3 | 8.5 | 8.3 |
| 169024 | mmu-miR-3960 | 1.2 | 3.4E-04 | 9.6 | 9.5 | 9.5 | 10.5 | 10.6 | 11.1 |
| 42572 | mmu-miR-154-3p | 1.2 | 2.3E-04 | 6.1 | 5.8 | 5.8 | 6.8 | 7.2 | 7.2 |
| 146172 | mmu-miR-1892 | 1.2 | 3.9E-04 | 8.2 | 8.1 | 8.0 | 9.6 | 9.0 | 9.2 |
| 169148 | mmu-miR-5130 | 1.2 | 2.6E-04 | 6.4 | 5.8 | 6.1 | 7.3 | 7.2 | 7.3 |
| 42707 | mmu-miR-294-5p | 1.2 | 2.0E-03 | 8.4 | 7.5 | 8.3 | 9.4 | 9.2 | 9.3 |
| 146081 | mmu-miR-1929-5p | 1.2 | 2.3E-04 | 6.5 | 5.9 | 6.1 | 7.4 | 7.5 | 7.4 |
| 148103 | mghv-miR-M1-4-3p | 1.2 | 4.2E-03 | 8.1 | 7.1 | 8.3 | 9.1 | 9.1 | 9.0 |
| 148531 | mmu-miR-544-5p | 1.2 | 3.4E-05 | 6.0 | 5.8 | 5.8 | 7.0 | 7.1 | 7.1 |
| 148533 | mmu-miR-1943-3p | 1.2 | 3.8E-05 | 7.3 | 7.3 | 7.5 | 8.6 | 8.7 | 8.6 |
| 148121 | mmu-miR-155-3p | 1.3 | 1.5E-04 | 6.4 | 5.9 | 6.0 | 7.4 | 7.4 | 7.2 |
| 168824 | mmu-miR-5100 | 1.3 | 3.5E-04 | 10.4 | 10.2 | 10.5 | 11.8 | 11.7 | 11.2 |
| 148644 | mmu-miR-551b-3p | 1.3 | 6.5E-05 | 5.9 | 5.6 | 6.0 | 7.0 | 7.2 | 7.1 |
| 27568 | mmu-miR-744-5p | 1.3 | 8.6E-05 | 10.1 | 10.5 | 10.4 | 11.8 | 11.5 | 11.7 |
| 29650 | mmu-miR-714 | 1.3 | 1.1E-04 | 9.4 | 9.4 | 9.3 | 10.9 | 10.4 | 10.8 |
| 145701 | mmu-miR-668-3p | 1.3 | 7.7E-04 | 6.8 | 5.8 | 6.3 | 7.6 | 7.7 | 7.7 |
| 148249 | mghv-miR-M1-6-5p | 1.4 | 4.6E-04 | 9.9 | 10.3 | 10.3 | 11.1 | 11.7 | 11.8 |
| 42659 | mmu-miR-290a-3p | 1.4 | 2.0E-02 | 9.0 | 7.2 | 8.8 | 9.6 | 9.8 | 9.6 |
| 148191 | mmu-miR-3081-3p | 1.4 | 9.9E-05 | 6.2 | 5.9 | 6.5 | 7.7 | 7.5 | 7.5 |
| 148175 | mmu-miR-1843a-3p | 1.4 | 1.1E-04 | 5.7 | 5.7 | 5.7 | 6.7 | 7.3 | 7.1 |
| 169051 | mmu-miR-5120 | 1.4 | 1.3E-04 | 6.3 | 5.8 | 6.2 | 7.3 | 7.7 | 7.5 |
| 168981 | mmu-miR-378b | 1.4 | 8.7E-06 | 5.6 | 5.7 | 5.6 | 7.0 | 7.1 | 7.1 |
| 148657 | mmu-miR-381-5p | 1.4 | 1.1E-04 | 5.9 | 6.0 | 6.3 | 7.2 | 7.7 | 7.5 |
| 148370 | mmu-miR-466n-3p | 1.4 | 1.9E-02 | 7.6 | 5.7 | 6.3 | 8.0 | 7.7 | 8.2 |
| 42619 | mmu-miR-709 | 1.4 | 1.5E-05 | 6.4 | 6.4 | 6.3 | 7.9 | 7.7 | 7.8 |
| 28450 | mmu-miR-291b-5p | 1.4 | 3.8E-04 | 7.0 | 6.2 | 6.9 | 8.2 | 8.0 | 8.2 |
| 145993 | mmu-miR-1899 | 1.4 | 4.8E-04 | 6.8 | 5.9 | 6.3 | 7.9 | 7.6 | 7.8 |
| 42730 | mmu-miR-423-3p | 1.4 | 4.7E-04 | 6.7 | 5.9 | 6.4 | 7.5 | 8.0 | 7.8 |
| 146070 | mmu-miR-1932 | 1.5 | 1.1E-05 | 6.0 | 5.8 | 5.9 | 7.2 | 7.3 | 7.5 |
| 148136 | mghv-miR-M1-14-3p | 1.5 | 5.4E-05 | 5.3 | 5.2 | 5.3 | 6.9 | 6.4 | 6.8 |
| 169060 | mmu-miR-3961 | 1.5 | 4.4E-04 | 10.1 | 10.8 | 10.6 | 11.6 | 12.1 | 12.2 |
| 148179 | mmu-miR-3095-3p | 1.5 | 3.8E-04 | 6.6 | 5.7 | 6.1 | 7.7 | 7.5 | 7.7 |
| 145897 | mmu-miR-92b-3p | 1.5 | 2.0E-05 | 6.1 | 5.7 | 5.8 | 7.3 | 7.3 | 7.5 |
| 169374 | mmu-miR-184-5p | 1.6 | 6.3E-03 | 7.9 | 6.4 | 8.0 | 8.9 | 9.0 | 9.0 |
| 145678 | mmu-miR-150-5p | 1.6 | 5.6E-03 | 8.8 | 7.1 | 8.5 | 9.6 | 9.7 | 9.8 |
| 148630 | mmu-miR-3472 | 1.7 | 6.6E-06 | 5.5 | 5.7 | 5.4 | 7.1 | 7.2 | 7.2 |
| 146111 | mmu-miR-767 | 1.7 | 1.7E-05 | 6.1 | 5.6 | 5.7 | 7.6 | 7.4 | 7.5 |
| 16681 | mmu-miR-721 | 1.7 | 1.1E-04 | 7.3 | 6.6 | 7.2 | 8.6 | 8.8 | 8.7 |
| 148037 | mmu-miR-363-5p | 1.7 | 3.6E-06 | 5.5 | 5.4 | 5.5 | 7.1 | 7.1 | 7.2 |
| 148416 | mmu-miR-3102-5p | 1.7 | 4.5E-05 | 6.5 | 6.1 | 6.2 | 8.1 | 7.6 | 8.1 |
| 145640 | mmu-miR-328-3p | 1.7 | 1.9E-05 | 6.2 | 5.7 | 5.9 | 7.5 | 7.6 | 7.7 |
| 146145 | mmu-miR-1895 | 1.7 | 5.7E-06 | 6.0 | 6.3 | 6.2 | 7.9 | 7.9 | 7.8 |
| 168738 | mmu-miR-5127 | 1.7 | 1.6E-04 | 6.9 | 6.1 | 6.9 | 8.2 | 8.4 | 8.4 |
| 19007 | SNORD3@ | 1.7 | 7.8E-06 | 7.5 | 7.9 | 7.8 | 9.3 | 9.4 | 9.5 |
| 11231 | mmu-miR-345-5p | 1.7 | 1.3E-04 | 6.4 | 5.5 | 5.8 | 7.7 | 7.5 | 7.7 |
| 169105 | mmu-miR-3963 | 1.7 | 6.1E-05 | 11.9 | 11.2 | 11.6 | 13.4 | 13.2 | 13.3 |
| 148210 | mmu-miR-3060-3p | 1.8 | 4.3E-04 | 6.7 | 5.6 | 5.9 | 7.8 | 7.9 | 7.9 |
| 42530 | mmu-let-7a-2-3p | 1.8 | 2.5E-06 | 6.3 | 6.4 | 6.4 | 8.0 | 8.2 | 8.2 |
| 148668 | mmu-miR-378a-3p | 1.8 | 8.0E-05 | 7.2 | 6.4 | 6.8 | 8.4 | 8.6 | 8.7 |
| 146201 | mmu-miR-1839-3p | 1.8 | 2.6E-04 | 6.3 | 5.3 | 5.7 | 7.6 | 7.7 | 7.4 |
| 148589 | mmu-miR-3109-5p | 1.8 | 5.8E-06 | 5.5 | 5.5 | 5.5 | 7.1 | 7.4 | 7.5 |
| 148579 | mmu-miR-3544-3p | 1.8 | 1.1E-05 | 6.4 | 5.9 | 6.2 | 7.9 | 8.0 | 8.0 |
| 42770 | mmu-miR-665-3p | 1.8 | 2.2E-05 | 6.4 | 6.3 | 5.8 | 8.1 | 7.9 | 8.0 |
| 148104 | mmu-miR-3092-3p | 1.8 | 3.4E-05 | 6.0 | 5.8 | 6.3 | 7.6 | 8.0 | 8.1 |
| 42878 | mmu-miR-882 | 1.8 | 1.6E-05 | 7.8 | 8.3 | 8.2 | 10.1 | 9.9 | 9.8 |
| 27575 | mmu-miR-711 | 1.8 | 1.7E-03 | 8.9 | 7.6 | 9.0 | 10.5 | 10.2 | 10.5 |
| 17489 | mmu-miR-710 | 1.9 | 1.3E-05 | 6.9 | 6.7 | 7.2 | 8.9 | 8.7 | 8.8 |
| 168797 | mmu-miR-3968 | 1.9 | 4.9E-04 | 6.8 | 5.6 | 6.7 | 8.5 | 8.1 | 8.2 |
| 17866 | mmu-miR-331-5p | 1.9 | 3.4E-05 | 6.3 | 5.6 | 6.1 | 8.1 | 7.8 | 7.9 |
| 17822 | mmu-miR-490-5p | 2.0 | 6.9E-06 | 6.3 | 5.9 | 6.3 | 8.0 | 8.2 | 8.3 |
| 27574 | mmu-miR-705 | 2.0 | 5.1E-05 | 5.4 | 5.2 | 6.0 | 7.7 | 7.6 | 7.3 |
| 148339 | mmu-miR-665-5p | 2.0 | 5.2E-03 | 7.6 | 5.9 | 7.9 | 9.0 | 9.2 | 9.1 |
| 148490 | mmu-miR-1224-3p | 2.1 | 3.8E-06 | 8.0 | 8.4 | 8.1 | 10.1 | 10.3 | 10.5 |
| 46636 | mcmv-miR-M23-1-5p | 2.1 | 2.3E-03 | 7.5 | 5.6 | 5.9 | 8.7 | 8.2 | 8.5 |
| 147366 | mmu-miR-320-5p | 2.1 | 6.1E-07 | 6.2 | 6.3 | 6.2 | 8.4 | 8.5 | 8.3 |
| 17632 | mmu-miR-691 | 2.2 | 1.9E-05 | 9.7 | 10.2 | 9.8 | 11.8 | 12.0 | 12.4 |
| 169291 | mmu-miR-5126 | 2.2 | 1.9E-06 | 6.1 | 5.8 | 6.1 | 8.2 | 8.1 | 8.4 |
| 148045 | mmu-miR-3094-3p | 2.2 | 6.8E-06 | 5.5 | 5.2 | 5.4 | 7.3 | 7.9 | 7.6 |
| 148100 | mmu-miR-1947-3p | 2.2 | 2.6E-05 | 12.0 | 11.1 | 11.3 | 13.8 | 13.7 | 13.7 |
| 42826 | mmu-miR-300-5p | 2.3 | 1.5E-05 | 12.3 | 11.5 | 11.6 | 14.2 | 14.0 | 14.0 |
| 145677 | mmu-miR-139-5p | 2.3 | 2.2E-05 | 5.5 | 5.5 | 5.6 | 7.3 | 8.2 | 8.1 |
| 28624 | mmu-miR-666-5p | 2.3 | 1.7E-05 | 6.0 | 5.4 | 5.7 | 8.3 | 7.7 | 8.0 |
| 169290 | mmu-miR-5617-5p | 2.4 | 5.0E-04 | 7.2 | 5.5 | 6.7 | 8.8 | 8.8 | 8.9 |
| 147701 | mmu-miR-491-3p | 2.4 | 4.4E-04 | 9.8 | 8.4 | 9.2 | 11.5 | 12.0 | 11.0 |
| 42694 | mmu-miR-485-3p | 2.4 | 3.9E-04 | 7.1 | 5.5 | 6.2 | 8.6 | 8.9 | 8.7 |
| 148128 | mmu-miR-3090-5p | 2.5 | 5.8E-06 | 6.4 | 5.7 | 6.2 | 8.6 | 8.4 | 8.7 |
| 146155 | mmu-miR-2137 | 2.6 | 3.6E-04 | 11.8 | 10.1 | 11.3 | 13.9 | 13.5 | 13.5 |
| 42811 | mmu-miR-542-5p | 2.6 | 3.9E-03 | 9.1 | 6.4 | 7.5 | 10.5 | 9.9 | 10.4 |
| 42702 | mmu-miR-30c-1-3p | 2.6 | 2.2E-06 | 6.3 | 5.6 | 5.9 | 8.6 | 8.5 | 8.5 |
| 42808 | mmu-miR-874-3p | 2.6 | 1.4E-04 | 6.7 | 5.3 | 5.5 | 8.6 | 8.4 | 8.4 |
| 146187 | mmu-miR-1941-3p | 2.7 | 5.3E-04 | 7.9 | 6.1 | 7.3 | 9.5 | 9.9 | 10.0 |
| 168688 | mmu-miR-1843b-3p | 2.7 | 1.2E-04 | 12.5 | 11.2 | 11.9 | 14.5 | 14.2 | 15.0 |
| 148212 | mmu-miR-3103-3p | 2.8 | 4.5E-06 | 9.3 | 9.1 | 9.2 | 11.5 | 12.1 | 12.3 |
| 168787 | mmu-miR-5114 | 2.9 | 1.6E-04 | 8.4 | 8.1 | 8.5 | 12.1 | 10.4 | 11.2 |
| 169364 | mmu-miR-3572-3p | 2.9 | 2.3E-05 | 6.9 | 5.9 | 6.4 | 8.9 | 9.6 | 9.4 |
| 168740 | mmu-miR-5113 | 3.2 | 1.2E-07 | 9.1 | 9.1 | 9.2 | 12.2 | 12.2 | 12.6 |
| 27855 | mmu-miR-763 | 4.0 | 1.4E-08 | 5.7 | 5.9 | 5.6 | 9.6 | 9.7 | 9.8 |
